# Neocentromeres Provide Chromosome Segregation Accuracy and Centromere Clustering to Multiple Loci along a *Candida albicans* Chromosome

**DOI:** 10.1371/journal.pgen.1006317

**Published:** 2016-09-23

**Authors:** Laura S. Burrack, Hannah F. Hutton, Kathleen J. Matter, Shelly Applen Clancey, Ivan Liachko, Alexandra E. Plemmons, Amrita Saha, Erica A. Power, Breanna Turman, Mathuravani Aaditiyaa Thevandavakkam, Ferhat Ay, Maitreya J. Dunham, Judith Berman

**Affiliations:** 1 Department of Genetics, Cell Biology and Development, University of Minnesota, Minneapolis, Minnesota, United States of America; 2 Department of Biology, Grinnell College, Grinnell, Iowa, United States of America; 3 Department of Biology, Gustavus Adolphus College, Saint Peter, Minnesota, United States of America; 4 Department of Genome Sciences, University of Washington, Seattle, Washington, United States of America; 5 Department of Microbiology and Biotechnology, George S. Wise Faculty of Life Sciences, Tel Aviv University, Ramat Aviv, Israel; 6 La Jolla Institute for Allergy and Immunology, La Jolla, California, United States of America; University of Connecticut, UNITED STATES

## Abstract

Assembly of kinetochore complexes, involving greater than one hundred proteins, is essential for chromosome segregation and genome stability. Neocentromeres, or new centromeres, occur when kinetochores assemble *de novo*, at DNA loci not previously associated with kinetochore proteins, and they restore chromosome segregation to chromosomes lacking a functional centromere. Neocentromeres have been observed in a number of diseases and may play an evolutionary role in adaptation or speciation. However, the consequences of neocentromere formation on chromosome missegregation rates, gene expression, and three-dimensional (3D) nuclear structure are not well understood. Here, we used *Candida albicans*, an organism with small, epigenetically-inherited centromeres, as a model system to study the functions of twenty different neocentromere loci along a single chromosome, chromosome 5. Comparison of neocentromere properties relative to native centromere functions revealed that all twenty neocentromeres mediated chromosome segregation, albeit to different degrees. Some neocentromeres also caused reduced levels of transcription from genes found within the neocentromere region. Furthermore, like native centromeres, neocentromeres clustered in 3D with active/functional centromeres, indicating that formation of a new centromere mediates the reorganization of 3D nuclear architecture. This demonstrates that centromere clustering depends on epigenetically defined function and not on the primary DNA sequence, and that neocentromere function is independent of its distance from the native centromere position. Together, the results show that a neocentromere can form at many loci along a chromosome and can support the assembly of a functional kinetochore that exhibits native centromere functions including chromosome segregation accuracy and centromere clustering within the nucleus.

## Author Summary

The accurate segregation of chromosomes during cell division is essential for maintaining genome integrity. The centromere is the DNA region on each chromosome where assembly of a large protein complex, the kinetochore, is required to maintain proper chromosome segregation. In addition, active centromeres exhibit a specific three-dimensional organization within the nucleus: the centromeres associate with one another in a clustered manner. Neocentromeres, or new centromeres, appear at new places along the chromosome when a native centromere becomes non-functional. We used a yeast model, *Candida albicans*, and isolated twenty instances in which neocentromeres had formed at different positions. All of these neocentromeres were able to direct chromosome segregation, but some had increased error rates. Like native centromeres, these neocentromeres cluster in the nucleus with the other active centromeres. This implies that formation of a neocentromere leads to reorganization of the three-dimensional structure of the nucleus so that different regions of the chromosome are in closer contact to regions of other chromosomes. Recent work suggests that approximately 3% of cancers may contain chromosomes with neocentromeres. Our observations that many neocentromeres have increased error rates provides insight into genome instability in cancer cells. Changes in chromosome copy number may benefit the cancer cells by increasing numbers of oncogenes and/or drug resistance genes, but may also sensitize the cells to chemotherapy approaches that target chromosome segregation mechanisms.

## Introduction

Genome stability requires accurate chromosome segregation. Faithful chromosome segregation requires the assembly of a kinetochore complex on the centromere DNA region of each chromosome. The kinetochore is a large complex of more than 100 proteins and is essential for the attachment of the spindle microtubules to each chromosome during cell division [1]. Defects in chromosome segregation accuracy can cause DNA damage and chromosome rearrangements as well as aneuploidy, an imbalance in the numbers of individual chromosomes [2,3,4].

In most eukaryotes, the mechanisms that specify centromeres and that direct kinetochore assembly to a particular chromosomal region are epigenetic, rather than strictly sequence-dependent. CENP-A, a variant histone H3 protein, is an essential component of centromeric chromatin (reviewed in [5]). In addition to the presence of CENP-A at centromeres, centromeric chromatin is marked by other histone modifications. For example, histone H3K9 methylation and other indicators of heterochromatin mark pericentromere regions in humans and many other species [5]. Hypoacetylation of histone H4 is associated with gene silencing and is observed at centromere chromatin in budding yeast [6]. In *Schizosaccharomyces pombe*, the kinetochore mediates silencing of marker genes within the central core of the centromere [7]. However, recent data suggest that despite the association of many repressive marks at centromeres, transcription of non-coding RNA within the central core of centromere sequences is required for normal centromere function (reviewed in [8]). Transcription at centromeres must be carefully regulated because transcriptional levels that are either too low or too high are detrimental to kinetochore assembly [5,9]. However, we do not know how these optimal transcriptional levels are maintained nor whether kinetochore assembly has a direct role in regulating transcription.

Recent work has highlighted a consistent feature of functional centromeres in many organisms including *S*. *cerevisiae*, *Drosophila* and humans: they cluster together within a specific region within the 3D organization of the nucleus [10,11]. Centromere clustering provides a defining feature of yeast centromeres that has been used to identify centromeres in fungi with uncharacterized centromeres [12,13]. In *S*. *cerevisiae* and *C*. *albicans*, centromere clustering to a single focus is dependent on kinetochore-microtubule interactions, as strains lacking kinetochore components such as the Dam1 complex have clustering defects [14,15,16,17]. In other organisms including *Drosophila* [10], mouse, and human, centromeres cluster to multiple nuclear locations [11]. In *Drosophila*, clustering requires nucleoplasmin-like protein (NLP) and the insulator protein CTCF [10]. Interestingly, in *Drosophila*, tethering of kinetochore proteins to a plasmid causes association with the clusters [10]. Also, interfering with clustering disrupts pericentric heterochromatin causing increased expression of pericentric repeats [10]. This suggests that centromere clustering also may be important in transcriptional regulation at centromeres.

The position of the centromere on a given chromosome is inherited, such that syntenic centromeric loci are detected in related species [18,19,20]. Although the position of a centromere is generally stable through many generations, chromosome rearrangements, deletions, or amplifications sometimes form acentric chromosome fragments. Neocentromeres that assemble *de novo* at DNA loci not previously associated with kinetochore proteins can restore the ability of an acentric chromosome fragment to segregate efficiently [21]. In rare cases, neocentromeres form in otherwise normal chromosomes, without physical deletion of the native centromere, presumably following inactivation of the native centromere through unknown mechanisms [22,23]. Evidence of centromere repositioning is observed rarely in human patients, but has been detected as “evolutionary new centromeres” in the genomes of humans, macaques, and donkeys [20,24]. Evolutionary new centromeres are repositioning events that become fixed in the population and are thought to be important steps in speciation [19,25]. More than 100 human neocentromere locations have been identified [26], with the majority found in patients with developmental disabilities and others found in cancer tissues [26]. For example, neocentromeres are characteristic cytogenetic features of well-differentiated liposarcomas [27]. Recent work has identified neochromosomes, many of which are predicted to have neocentromeres, in approximately 3% of cancers [28].

Several model systems have been developed to study neocentromere formation and function including *Drosophila* [29], *S*. *pombe* [30,31], *C*, *albicans* [32,33], and chicken cells [34]. Neocentromere locations in *Drosophila* and *S*. *pombe* are limited to specific chromosomal domains. For example, neocentromeres in *Drosophila* have been identified at pericentric regions [29], and mature neocentromeres in *S*. *pombe* form most frequently at subtelomeric regions and require adjacent heterochromatin for functionality [31]. Neocentromeres in humans and in chicken DT40 cells localize to diverse positions, many of which lack adjacent heterochromatin [34]. Thus, the range of possible neocentromere positions changes in different systems.

*C*. *albicans* has been established as a model for neocentromere formation. The small, regional centromeres of *C*. *albicans* all have unique DNA sequences of approximately 3-5kb bound by CENP-A [35]. Several centromeres, most predominately centromere 5 (*CEN5*), are flanked by inverted repeat sequences unique to that centromere [32]. Following deletion of native centromere DNA sequences, functional kinetochores assemble, evidenced by the appearance of CENP-A and other kinetochore proteins at new loci [32,33]. Neocentromeres also specify early replication timing, similar to native centromeres [36]. Neocentromeres can form either proximal or distal to the native centromere [32,33]. Neocentromere positions are inherited from one generation to the next, but neocentromere positions are less stable than native centromere positions. At low frequency, neocentromere positions shifted locally as detected by reversible silencing of a *URA3* gene in proximal neocentromere strains. Additionally, in one transformant from Ketel *et al*., the isolate was saved prior to neocentromere position stabilization and multiple neocentromere positions were isolated from a single transformant [32].

In most systems it has been difficult to compare the function of neocentromeres to native centromeres. In humans, some neocentromeres appear to be more prone to chromosome segregation errors than native centromeres. One characterized human neocentromere also has defects in the localization of Aurora B kinase, an essential regulator of kinetochore-microtubule attachments, and in error correction [37]. Neocentromere mosaicism, defined as the presence of the neocentromere in a subset of somatic cells, suggests that the chromosome carrying the neocentromere was lost in a subpopulation of the cells. Based on the available data in humans, it is not clear if the mosaic neocentromeres are due to processes related to the formation of the neocentromere, selective disadvantages of maintaining the neocentric chromosome, and/or defects in segregation accuracy of the neocentromere [26]. Importantly, other neocentromeres are found consistently in all patient tissues and appear to segregate accurately [26]. The possibility that different neocentromere loci have different levels of chromosome segregation accuracy is intriguing, but technical issues, such as differences in genetic background between individuals and difficulty in quantitating chromosome segregation, complicate rigorous comparisons of human neocentromeres. Using *C*. *albicans* as a model system allows us to eliminate both of these obstacles. First, all *C*. *albicans* neocentromeres can be isolated from the same parental strain, which reduces the effect of genetic diversity. Second, a sensitive method to quantify small to moderate increases in chromosome loss is readily available, based upon selection for loss of the *URA3* marker gene by growth of cells on 5-fluorourotic acid (5-FOA) [38].

In this work, we characterized twenty neocentromere loci on *C*. *albicans* chromosome 5 (Chr5). These neocentromeres were assembled at intergenic regions as well as at loci containing ORFs, where the neocentromere repressed ORF transcription. Some, but not all neocentromere strains had higher chromosome loss rates than strains with native centromeres. Thus, as in humans, neocentromeres in *C*. *albicans* can have variable degrees of functionality at different loci. Finally, neocentromere formation drives reorganization of interchromosomal interactions, such that the functional neocentromere, like native centromeres on unperturbed chromosomes, clusters with active native centromeres on other chromosomes. This indicates that the three-dimensional (3D) organization of centromere clustering is a dynamic process and is dependent upon epigenetic kinetochore function rather than upon DNA sequence in *C*. *albicans*.

## Results

### Identification of additional distal neocentromere positions

Strains that survive deletion of the 7.6kb centromere region on Chr5, which includes the central core and both flanking inverted repeat sequences, form neocentromeres at locations proximal to (within 4kb of the deleted region) or more distal to the native centromere locus [32,33]. In Ketel *et al*., proximal neocentromere strains all were centered at 464.5kb and two independent transformants resulted in four distal neocentromeres at loci along the length of Chr5 [32]. Thakur and Sanyal (2013) also deleted a 7.2kb region of *CEN5* and all 6 neocentromeres characterized were centered nearby the deleted sequence at ~459kb and ~478kb [33]. To ask if neocentromere loci are limited to specific chromosome arm regions, we isolated additional transformants in which *CEN5* sequences were replaced with *URA3* (S1 Fig). Combined with the transformants described in Ketel *et al*. [32], ~ 50% of transformation events resulted in distal neocentromeres (S1 Fig). Previously, three of the four distal neocentromere positions were isolates obtained from a single transformant stock from neocentromere movement or displacement following sorbose treatment, a nutrient stress condition resulting in high rates of homozygosis of Chr5 [32,39]. In addition to neocentromere movement following stress, colony purification of a single transformant revealed sub-clones with different neocentromere positions. Subsequently, some of these neocentromeres were detectable in bulk analysis of the original stored stock. Thus, we hypothesized that, immediately following transformation, neocentromeres may be unstable and that subpopulations of a transformant colony might contain different neocentromere loci. Therefore, in addition to testing multiple colonies from the new transformants, we also isolated and characterized additional single colonies from previously published transformants with distal neocentromeres.

**Fig 1:**
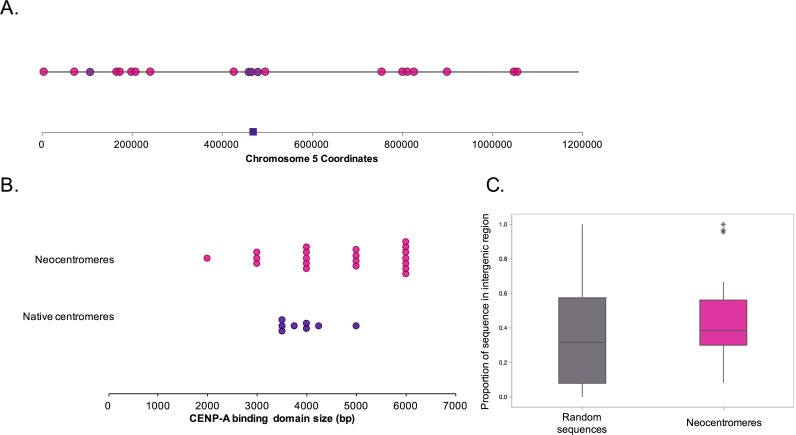
Neocentromere positions and CENP-A binding domain sizes. A. Schematic of neocentromere positions identified in this work and in Ketel *et al*. [32]. A pink circle indicates each non-overlapping neocentromere isolated once. Plum colored circles indicate neocentromeres found in more than one transformant. The dark purple square indicates the native centromere location on Chr5. B. CENP-A binding domain size was estimated by anti-CENP-A ChIP followed by hybridization to a tiling microarray or high-throughput sequencing. Start and stop coordinates of centromeres (dark purple) and neocentromeres (pink) were estimated to the nearest 250bp for all samples. C. Box plot of the proportion of the neocentromere genomic regions (pink) located within intergenic regions compared to size-matched random genomic regions (gray). * indicate outliers.

We then identified the positions of the neocentromeres by chromatin immunoprecipitation (ChIP) with anti-CENP-A antibodies on the newly isolated strains as well as on additional isolates of the previous transformants (S2 Fig). In addition, the neocentromere position originally identified at ~170kb in strains YJB10779 and YJB10780 [32] was mapped at higher resolution to two adjacent, non-overlapping neocentromere positions centered at 173.5kb and 166kb, respectively (S3 Fig). One distal neocentromere position was identified in isolates from two independent transformants in our lab, and two neocentromere positions overlapped with the neocentromere positions identified in Thakur and Sanyal [33]. All other neocentromere positions were observed only from a single transformant, indicating that the screen for neocentromere positions has not yet saturated all possible loci, but that positions capable of supporting neocentromere function are likely not infinite. Together, this brings the total number of neocentromere positions to twenty including the proximal neocentromere position at 464.5kb (Fig 1A, S1 Table).

### DNA sequence features of neocentromeres

Using these twenty neocentromeres, we searched for DNA sequence features that could significantly distinguish neocentromere loci from native centromeres and/or from all other regions of Chr5. While neocentromere regions are more variable in size than native centromere regions, no significant differences were found between the size of the CENP-A bound DNA sequence in neocentromeres and native centromeres (unpaired t-test, p>0.05) (Fig 1B). The GC% values ± SD for neocentromeres (33.7 ± 3.3%), native centromeres (35.0 ± 1.0%), or size-matched random DNA regions on chromosome 5 (32.6 ± 2.2%) also were not significantly different (one-way ANOVA, p>0.05). Skew inversions for G/C distribution were previously identified at *C*. *albicans* centromeres as fossils of the long-term presence of early origins of DNA replication (S4A Fig) [36]. This is thought to occur because leading strands and lagging strands cause biased rates of C→G transversions and this bias would ‘flip’ at a constitutive origin where leading strands emerge in opposite directions [40]. Importantly, neocentromere formation promotes early/efficient replication initiation [36], yet neocentromeres have not been constitutive early origins over the long time scales necessary to accumulate skew inversion patterns (where the skew level crosses the X-axis 0 line, S4 Fig); consistent with this, we did not identify consistent G/C skew inversion patterns at the neocentromere loci (S4B Fig) or at random loci (S4C Fig). Native centromeres in *C*. *albicans* are associated with three different types of repeat elements: inverted repeats, tandem repeats, and transposon-associated repeats (S5A Fig) and repeat elements are important in *de novo* kinetochore assembly on a plasmid in the closely related species *C*. *tropicalis* [41]. Yet the distance between the center of each neocentromere and the closest repeat element (mean distance ± SEM, 3622 ± 745bp) was not significantly different from the corresponding distances of random loci and repeat elements on Chr5 (4165 ± 1013bp) (unpaired t-test, p>0.05) (S5B Fig).

Early observations with small numbers of distal neocentromeres suggested that neocentromeres formed in intergenic regions [32]. However, more detailed mapping and the increased number of neocentromere strains revealed neocentromeres that mapped within ORFs as well. Indeed, neocentromere regions and random sequences were similarly likely to be intergenic versus genic (unpaired t-test, p>0.05) (Fig 1C). ORFs overlapped with the neocentromere position by at least 100bp for 19 of the 20 neocentromere positions (S1 Table). Using more conservative criteria, 17 of 20 neocentromeres have >500bp 5’ ORF overlap, >1000bp 3’ ORF overlap, or overlap of the entire ORF.

### Neocentromere formation represses transcription

The relationship between kinetochore assembly and transcription is complex, as low transcription levels benefit centromere function and high levels of transcription are incompatible with the presence of a functional kinetochore [8,9]. We next asked if ORFs with the potential to become neocentromeres are transcribed under standard laboratory conditions, by analyzing previously published RNA-seq data for *C*. *albicans* grown in YPD medium at 30°C [42]. *C*. *albicans* were grown in YPD immediately preceding the centromere deletion event that promoted neocentromere formation, so these transcription levels likely represent the transcription state of the cell prior to the induction of neocentromere formation. In the Bruno *et al*. data set, transcripts in YPD grown cells ranged from -4.6 to 13.6 (on a log2 scale of reads per kilobase per million mapped reads (RPKM)). Interestingly, 13 of 20 neocentromere positions have transcripts within the neocentromere DNA region at levels equal to or greater than the median expression level of all ORFs in YPD in the RNA-seq data set (S1 Table). This suggests that neocentromeres assembled at ORFs that are normally transcribed in the context of native chromosomes.

**Fig 2:**
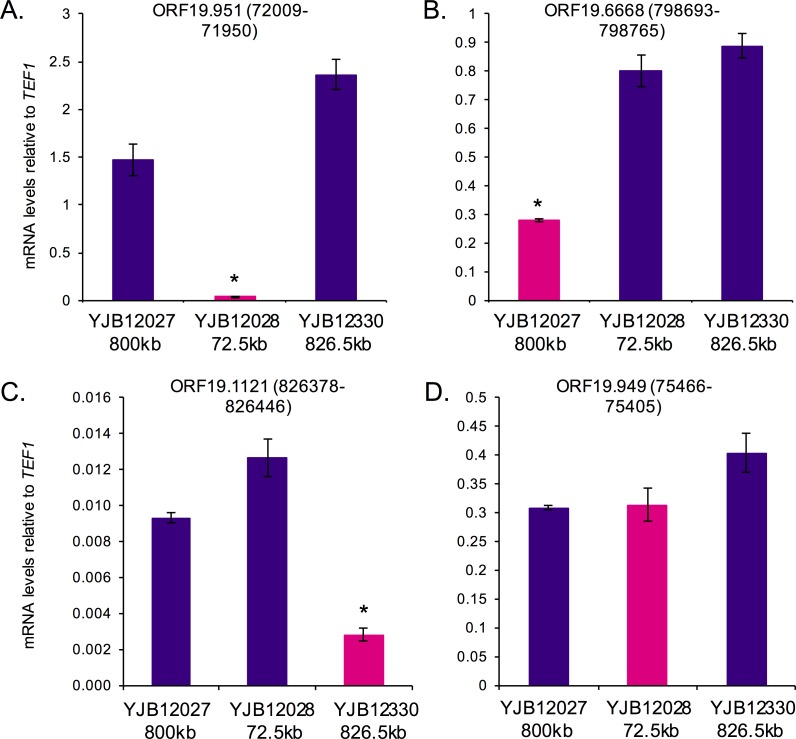
Transcriptional activity is repressed following neocentromere formation. Homozygous neocentromere strains YJB12027 (800kb center), YJB12028 (72.5kb center), and JYB12330 (826.5kb center) were grown in YPAD for 4 h. mRNA levels for *ORF19*.*951* (A), *ORF19*.*6668* (B), *ORF19*.*1121* (C) and *ORF19*.*949* (D) relative to the reference gene *TEF1* were measured by qRT-PCR. Data shown are mean ± SEM of 3 biological replicates. * p<0.01 by ANOVA and Tukey post-tests.

To ask if neocentromeric chromatin suppresses the expression of genes within the CENP-A binding region as has been observed with marker genes at native centromeres in *S*. *pombe* [32,43], we measured transcription levels of native genes within defined neocentromere regions. We conducted qRT-PCR in strains with an active neocentromere at the given locus, and at least two other strains with neocentromeres formed at other Chr5 loci. Importantly, for a given region, transcript levels at an active neocentromere were lower than those at the same loci in strains without the neocentromere at that locus (Fig 2A–2C and S6A–S6F Fig). By contrast, a neighboring transcript just outside the CENP-A binding region (Fig 2D), a transcript with the promoter >500bp from the CENP-A binding region (S6G Fig), and a transcript of a gene on a different chromosome (S6H Fig) showed no detectable difference in expression among three neocentromere strains strains. Thus, active neocentromeres suppress transcription at loci where they are assembled.

### Neocentromeres confer different degrees of chromosome segregation accuracy

**Fig 3:**
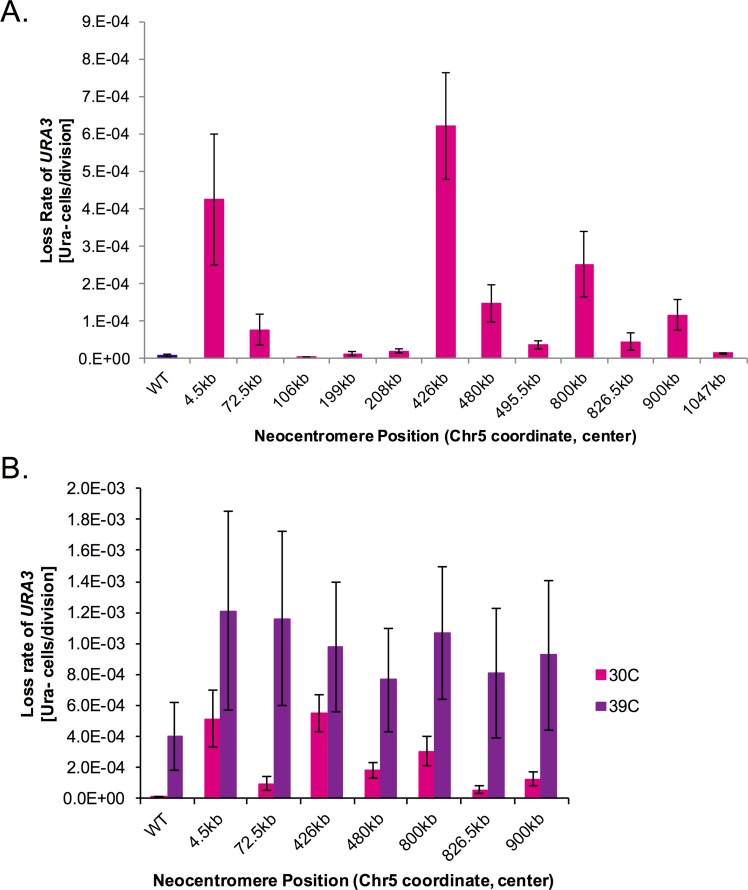
Neocentromere strains have different *URA3* loss rates. A. Fluctuation analysis of loss of *URA3* in control (*INT1/int1Δ*::*ura3*) (dark purple) and neocentromere *(CEN5/cen5Δ*::*ura3*) (magenta) strains. Cultures of each strain were grown in YPAD for 24 h at 30°C. Loss of *URA3* was quantified by plating cells on non-selective media (YPAD) and on media containing 5-FOA to select for loss of *URA3*. Colony counts were used to calculate the rate of loss per cell division. Results are the mean ± SEM of the rates calculated from at least 3 experiments, each with 8 cultures per condition. p<0.01 for strain differences by ANOVA. B. Cultures of each strain were grown in YPAD for 24 h at 30°C (magenta) or 39°C (purple). Loss of *URA3* was quantified by plating cells on non-selective media and on media containing 5-FOA to select for loss of *URA3*. Colony counts were used to calculate the rate of loss per cell division. Results are the mean ± SEM of the rates calculated from at least 3 experiments, each with 8 cultures per condition. p<0.01 for heat treatment differences and p>0.05 for heat*strain interaction by two-way ANOVA.

In humans, different neocentromeres appear to have different degrees of chromosome segregation accuracy (reviewed in [26]). To directly test whether different neocentromeres have different chromosome segregation accuracy, we compared chromosome segregation by following the loss of *URA3* in 12 heterozygous neocentromere strains where one copy of Chr5 maintains the native centromere and the other copy of Chr5 contains the neocentromere and the *URA3* marker. The *URA3* loss rate for the native centromere strain was approximately 1.0e-05 (Fig 3A). *URA3* loss rates for neocentromere strains ranged from approximately 4.6e-06 to 6.2e-04 (Fig 3A). Thus, some neocentromere strains had *URA3* loss rates very similar to the native centromere strain, while others had increased *URA3* loss rates of up to 60-fold higher than the native centromere strain. ANOVA analysis indicated that different neocentromere positions have statistically significant differences in *URA3* loss rate (p<0.01).

*URA3* loss is observed as the combined consequence of chromosome loss, shorter range recombination events and loss-of-function mutations in the *URA3* gene, which can be distinguished by SNP-RFLP analysis of markers on both arms of Chr5. In the seven neocentromere isolates with the highest *URA3* loss rates, loss of heterozygosity across all markers tested on Chr5 was elevated compared to the homozygosis of markers observed in strains with segregation driven by native centromere loci [44] (S2 Table). Thus, *URA3* was primarily lost via increased whole chromosome loss (homozygosis of all tested Chr5 SNP-RFLP markers), rather than an increase in recombination events (homozygosis of only some of the markers along Chr5) in neocentromere strains.

**Fig 4:**
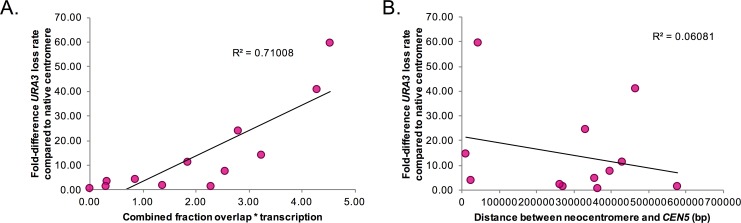
Neocentromere chromosome loss rate correlates with transcriptional activity, but not chromosomal position. A. The fold-difference in *URA3* loss rate between the mean rate for the native centromere strain and the mean rate of each neocentromere strain was plotted as a function of the fraction of the neocentromere CENP-A bound region that includes ORFs multiplied by the RNA-seq transcriptional measurement on a log2 scale of RPKM. Correlation between these two variables was high (r^2^ = 0.71). B. The fold-difference in *URA3* loss rate between the mean rate for the native centromere strain and the mean rate of each neocentromere strain was plotted as a function of the distance between the neocentromere position and the native centromere. Correlation between these two variables was very low (r^2^ = 0.06).

We next asked whether neocentromere function, measured as chromosome segregation accuracy, correlated with any of the other characteristics of neocentromere loci. No significant correlations were found for chromosome loss rates (Fig 3) relative to neocentromere length or the distance between the neocentromere and the nearest repeat element (S7A and S7B Fig). There was a slight positive correlation between chromosome loss rates and the fraction of the CENP-A binding region containing ORF sequences (S7C Fig). The correlation was much stronger when the fraction of ORF overlap and the RNA-seq transcription data were combined to estimate total transcriptional activity in the region prior to neocentromere formation (R^2^ = 0.71). Higher transcriptional activity correlates positively with higher chromosome loss (Fig 4A). This indicates that better neocentromere function is associated with DNA positions that normally have lower levels of transcription.

Certain types of stress, such as exposure to high temperature, elevate whole chromosome loss rates in *C*. *albicans* [44,45]. As expected, growth at 39°C significantly increased *URA3* loss rates (p<0.01) compared to growth at 30°C for neocentromeres as well as for native centromeres (Fig 3B). Again, whole chromosome loss was the likely mechanism based upon SNP-RFLP analysis of several markers (S2 Table). Interestingly, the fold-change in *URA3* loss between 30°C and 39°C was lower for the neocentromere strains, perhaps because chromosome loss rates were already elevated relative to the native centromeres at 30°C.

Specific chromatin modification patterns are required for accurate chromosome segregation. In *Saccharomyces cerevisiae*, nicotinamide treatment inhibits NAD^+^ histone deacetylation by Sir2-family proteins, resulting in centromere dysfunction [6]. Pericentromeric regions in *C*. *albicans* have elevated levels of H4K16 acetylation relative to the central core region [46]. Treatment with 2mM nicotinamide increased the relative level of H4K16 acetylation within the centromeric central core (S8A Fig). Thus, we asked if 2mM nicotinamide affected Chr5 loss rates in *C*. *albicans* with normal *CEN5* or different Chr5 neocentromeres. Overall, loss rates for native centromere strains and for 6 of 7 neocentromere strains increased with nicotinamide exposure (ANOVA, p<0.01), measured as *URA3* loss rates (S8B Fig). Treatment with 100μM nocodazole, an inhibitor of microtubule polymerization, also increased *URA3* loss rates for all tested neocentromeres (S9 Fig). In both drug treatments, the majority of the Ura^-^ isolates had SNP-RFLP markers indicating whole chromosome loss (S2 Table). Similar to heat stress, the fold-change between no drug and nicotinamide treatment or nocodazole treatment was less for the neocentromere strains, perhaps due to the higher initial chromosome loss rates. Together these results indicate that neocentromere strains are not hypersensitive to factors that disrupt centromere function. Nonetheless, the same types of stresses and drugs that affect native centromeres affect most neocentromere strains.

Neocentromere formation can occur along the entire length of the chromosome in *C*. *albicans* (Fig 1A) including, but not restricted to positions proximal to the native centromere [32,33,34]. Importantly, this data clearly refutes the suggestion that only neocentromeres close to the native centromere position are truly functional in *C*. *albicans* [33]. Furthermore, a comparison of *URA3* loss rates at different neocentromeres found no correlation between chromosome loss and distance of the neocentromere from the native centromere position (Fig 4B). Therefore, neocentromeres both close and far from the native centromere position can be highly functional in *C*. *albicans*.

### Neocentromeres cluster with centromeres based on the presence of a functional kinetochore

Yeast centromeres cluster in a single nuclear location, providing a driving force for the nuclear organization in fungi [47,48]. Mapping of chromosomal interactions with the chromatin conformation capture assay Hi-C is an effective way to identify functional centromere regions based on their 3D colocalization with one another [12,13,49,50]. Therefore, we tested the hypothesis that centromere clustering is an epigenetic feature of centromere function and is independent of physical or sequence-based features of the native centromere position. This hypothesis predicts that neocentromere formation would reorganize interchromosomal interactions, bringing the newly formed neocentromere on one chromosome with the native centromeres on the remaining chromosomes. We used Hi-C to identify all chromatin interactions for a strain with all centromeres at native locations and for two strains with an active neocentromere at different positions: one with homozygous neocentromeres centered near the left telomere at 4.5kb (YJB10777) and one with homozygous neocentromeres centered at 166kb (YJB10780). We mapped Hi-C data from these three strains to the *C*. *albicans* reference genome and further processed the mapped read pairs to produce raw and normalized Hi-C contact maps (Methods).

**Fig 5:**
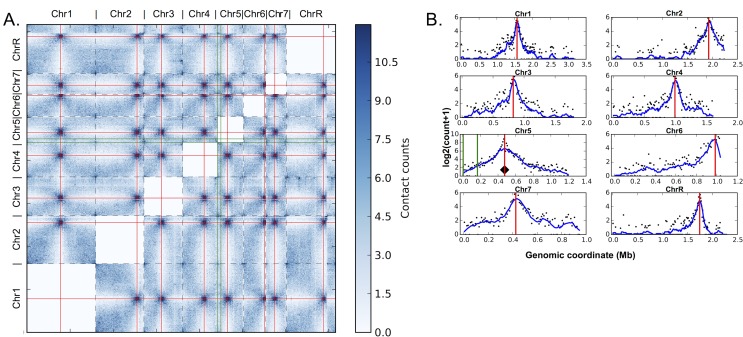
Centromere clustering is a feature of active centromeres in *C*. *albicans*. Red lines mark centromeres. Green lines indicate neocentromere positions. Black diamond indicates the viewpoint for the plotted interaction profiles. A. Heatmap of genome-wide interchromosomal interactions from the Hi-C data of the wild type strain with all active centromeres at their native positions. Normalized contacts counts are shown in increasing intensity of blue. Borders of chromosomes are shown with dashed lines. B. Virtual 4C plots from the 10kb sequence surrounding the center of native CEN5 showing log-scaled Hi-C contact counts for all *C*. *albicans* chromosomes in the wild type strain.

**Fig 6:**
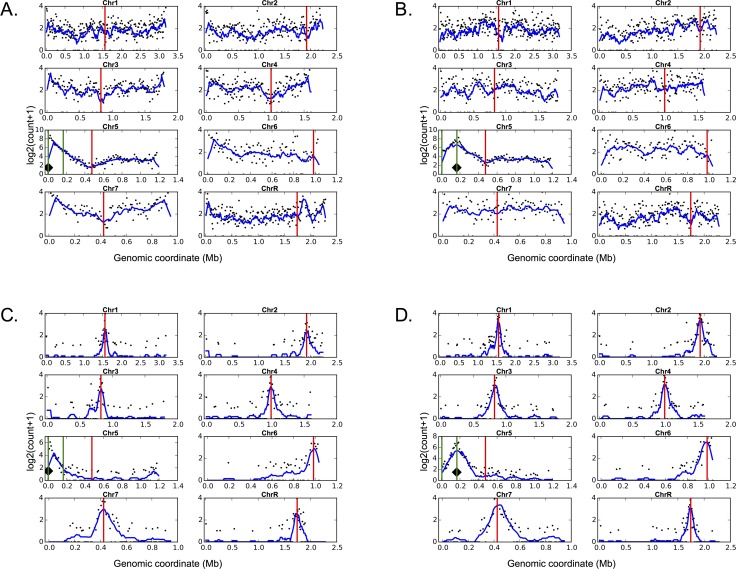
Neocentromere formation results in epigenetic activation of centromere clustering. Red lines mark centromeres. Green lines indicate neocentromere positions. Black diamond indicates the viewpoint for the plotted interaction profiles. A. Virtual 4C plots from the 10kb sequence surrounding the 4.5kb neocentromere region showing log-scaled Hi-C contact counts for all *C*. *albicans* chromosomes in the wild type (non-neocentromere) strain. B. Virtual 4C plots from the 10kb sequence surrounding the 166kb neocentromere region showing log-scaled Hi-C contact counts for all *C*. *albicans* chromosomes in the wild type (non-neocentromere) strain. C. Virtual 4C plots from the 10kb sequence surrounding the 4.5kb neocentromere region showing log-scaled Hi-C contact counts for all *C*. *albicans* chromosomes in YJB10777 (4.5kb neocentromere) strain. D. Virtual 4C plots from the 10kb sequence surrounding the 166kb neocentromere region showing log-scaled Hi-C contact counts for all *C*. *albicans* chromosomes in YJB10780 (166kb neocentromere) strain.

In the wild-type *C*. *albicans* strains, native centromeres on all chromosomes clustered with one another in 3D (Fig 5). This clustering was evident from the strong enrichment of centromere interactions, apparent both in the raw (data available via the Short Read Archive) and in the normalized interchromosomal contact maps (Fig 5A), as well as from the peaks of interchromosomal interactions near all centromere pairs when the native *CEN5* location was used as the interaction probe to create virtual 4C plots (Fig 5B). The peaks between all centromere pairs were conserved when any other native centromere was used as the probe (S10 Fig). These interchromosomal interaction patterns are similar to what has been seen in other yeast species [49]. Next, centromere positions were predicted solely from the Hi-C data using the Centurion algorithm [49]. For data from the wild-type strain, six of eight centromere midpoint predictions fell within the boundaries and the remaining two were within 2kb of native centromere positions estimated by CENP-A ChIP mapping (S3 Table). This result suggests that the generated Hi-C data provides sufficient information to accurately locate the centromeres. In the wild-type strain data (prior to neocentromere formation), regions where neocentromeres could form did not exhibit strong interactions with other centromeres. The DNA region near 4.5kb showed local Hi-C interactions with neighboring regions on Chr5 and some interactions with other telomeric regions, but not with centromeres in the wild-type strain (Fig 6A). Similarly, the DNA region near 166kb showed local interactions with neighboring regions on Chr5, but not with native centromeres in the wild type (Fig 6B).

**Fig 7:**
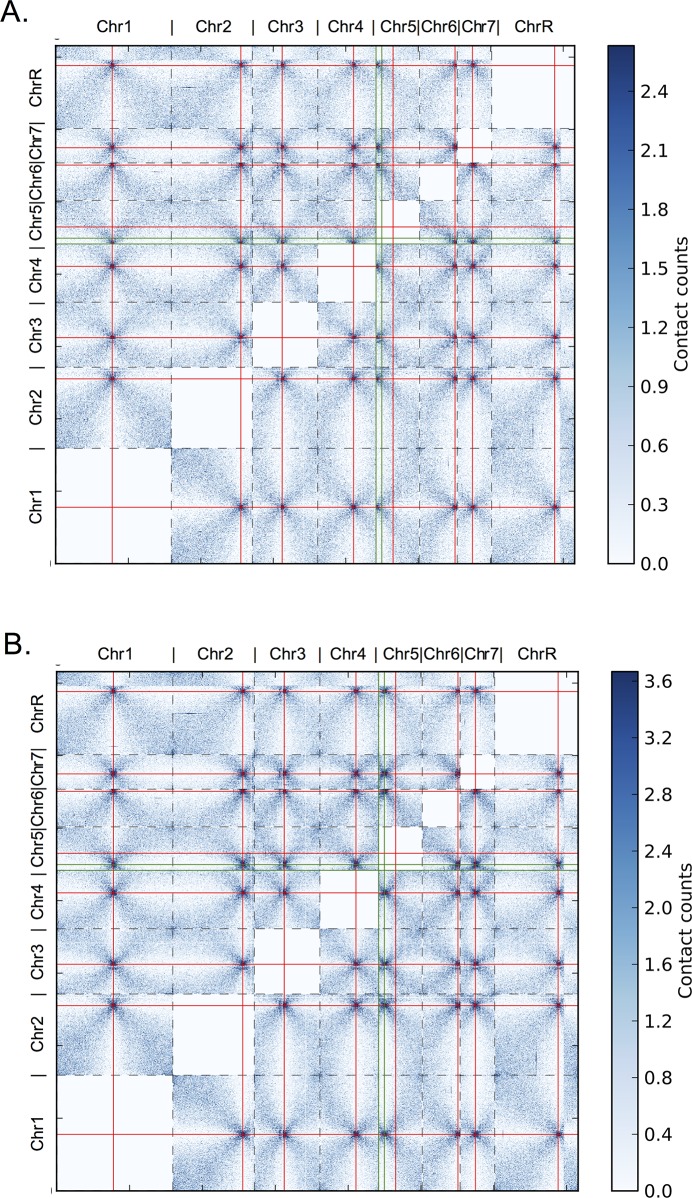
Neocentromere formation results in genome-wide shift of interchromosomal interactions from the native *CEN5* region to the neocentromere region. In the heat maps, normalized contacts counts are shown in increasing intensity of blue. Borders of chromosomes are shown with dashed lines. Red lines mark centromeres. Green lines indicate neocentromere positions. A. Heatmap of genome-wide interchromosomal interactions from the Hi-C data of the YJB10777 strain with the active centromere at the 4.5kb neocentromere position. B. Heatmap of genome-wide interchromosomal interactions from the Hi-C data of the YJB10780 strain with the active centromere at the 166kb neocentromere position.

In contrast, in the two strains with homozygous active neocentromeres centered at 4.5kb and 166kb (in which native *CEN5* had been deleted), the neocentromere regions clustered with centromeres on other chromosomes. In the strain with the neocentromere at the 4.5kb locus, the DNA region ~ 4.5kb from the left telomere interacted with all other centromeres (Fig 6C). Similarly, the homozygous active neocentromere near 166kb interacted with the native centromere loci of all other chromosomes (Fig 6D). Furthermore, genome-wide analysis of interactions demonstrated that the native centromere region on Chr5 did not interact with other centromeres and that centromeres on all chromosomes established reciprocal interactions with the neocentromere positions (Fig 7 and S11 Fig). In addition, the Centurion algorithm accurately predicted the neocentromere positions (as previously determined by chromatin immunoprecipitation with the centromere-specific histone CENP-A) further demonstrating that neocentromere formation was accompanied by an overall change in chromosome organization (S3 Table). Thus, neocentromeres acquire an important phenotype that is characteristic of centromeres—the ability to cluster with native centromeres on all other chromosomes.

## Discussion

Many loci on Chr5 are capable of supporting neocentromere formation. The most frequent position is immediately adjacent to the native centromere [32]. This proximal neocentromere position exhibits reversible silencing of the *URA3* marker used to delete the centromere and inverted repeat sequences [32]. Indeed, ~50% of transformants tested exhibited *URA3* silencing (11/22), indicative of proximal neocentromere formation (S1 Fig) [32]. Silencing was only observed when the center of the CENP-A region was within 2kb of the deleted sequence. In addition, three neocentromere positions did not exhibit *URA3* silencing, but were located within 30kb of the native centromere position (S1 Table). The increased likelihood of neocentromere formation near the native centromere, compared to any other single location on Chr5, is similar to neocentromere formation tendencies on chromosome Z in chicken DT40 cells where 76.2% of neocentromeres formed near the native centromere position [34]. The regions near the native centromere are potentially enriched for non-centromeric CENP-A in a “CENP-A cloud” [34]. Furthermore, these centromere proximal regions are in closer 3D proximity to one another in the wild-type nucleus (Fig 5), which may increase the opportunity for closer sequences to capture kinetochore proteins upon their release when the native centromere is deleted.

Importantly, we characterized sixteen neocentromere loci across the chromosome with kinetochore assembly occurring more than 30kb from the native centromere (S1 Table) [32]. These neocentromeres are functional, not only in chromosome segregation but also in centromere clustering, and thus are clearly able to serve as active neocentromeres. This is in contrast to the suggestions of Thakur and Sanyal (2013), who analyzed 6 neocentromeres on Chr5, and argued that *C*. *albicans* only forms neocentromeres near the native centromere [33]. Clearly, if sufficient numbers of neocentromeres are collected and analyzed, a significant proportion of them form active neocentromeres at distal positions on Chr5 greater than 30kb from the native centromere.

Once formed, neocentromeres on all regions of the chromosome promote chromosome segregation. Six of twelve tested neocentromeres had loss rates within 5-fold of native centromeres, further demonstrating that many distal neocentromere loci are active and functional (Fig 3). The functional similarity between distal neocentromeres and native centromeres is reinforced by their similar increase in chromosome loss rates in response to stressors such as high temperature, NAD-dependent histone deacetylase inhibitors, and the microtubule-destabilizing agent nocodazole (Fig 3, S8 Fig and S9 Fig). Strikingly, some neocentromeres conferred more accurate chromosome segregation than others (Fig 3) and this correlated with the estimated total transcriptional activity in the region prior to neocentromere formation (Fig 4). Specifically, higher transcriptional activity correlates positively with higher chromosome loss. This indicates that chromosome segregation is more efficient in regions with lower native transcription levels. Consistent with this idea, in *S*. *cerevisiae*, low levels of transcription are compatible with centromere function, high levels of transcription are disruptive [9,51]. We posit that kinetochore assembly on DNA competes with transcription initiation and thus, the stronger the affinity of the transcription machinery for the DNA, the weaker, or less functional is the assembled kinetochore on the neocentromere.

Neocentromere formation results in transcriptional repression of ORFs within the centromeric chromatin, further supporting the model that kinetochore assembly competes with transcription initiation. Transcriptional repression is evident at proximal neocentromeres, where the presence of *URA3* facilitates the detection of reversible silencing [32], but is also clear from analysis of transcript levels by qRT-PCR (Fig 2). In Shang *et al*., repression of a gene located within the CENP-A boundaries of a single neocentromere was found to be repressed following neocentromere formation [34]. Our data showing repression of nine different genes at five *C*. *albicans* neocentromeres (Fig 2 and S6 Fig) supports the idea that transcriptional repression is a conserved feature of neocentromeres. Because the inhibition of transcription was limited to the CENP-A bound region and did not extend to neighboring genes (Fig 2 and S6 Fig), we propose that CENP-A recruitment to neocentromeres, and the resulting chromatin structure and kinetochore complex assembly, hinders the transit of transcription complexes through the region.

Most neocentromere loci were initiated by deleting one of the two *CEN5* copies, such that in the original isolates only one allele would be associated with CENP-A and repressed, while the other would be expressed. Interestingly, four ORFs within neocentromere CENP-A binding regions have homologs in *S*. *cerevisiae* that are essential for growth under normal laboratory conditions: orf19.3166, orf19.3161, orf19.4221, and orf19.4230. For three of these neocentromere loci, we were unable to isolate homozygous centromere deletion strains with CENP-A assembled on these genes suggesting that neocentromere formation in regions with putative essential genes can only occur on one allele. The fourth putative essential gene (orf19.3161) is found within the proximal neocentromere position that is positionally unstable as seen by reversible silencing of *URA3*, perhaps to allow access of RNA polymerase within the region [32]. We suggest that it may not be possible to form functional neocentromeres on both copies of an essential gene, as it would reduce transcription to levels that would be detrimental to growth and survival.

Importantly, the data clearly reveals that distal neocentromeres direct 3D centromere clustering like native centromeres (Fig 5, Fig 6 and Fig 7) and independent of their distance to the native centromere location. Indeed, not only does the distance of the neocentromere from the deleted native centromere not correlate with neocentromere chromosome segregation function (Fig 4), it also does not appear to affect centromere clustering. Thus, our study of neocentromeres demonstrates, for the first time, that centromere clustering, which has been observed in many fungi and can be used to identify functional centromeres, is an epigenetic feature of an active centromere and is independent of DNA sequence or chromosomal context. Importantly, the neocentromere centered at 4.5kb clearly clustered with other centromeres despite having the second-highest chromosome loss rate, albeit less than 0.05% (Fig 3). Thus, even neocentromeres at the lower end of the chromosome segregation function scale still recapitulate a remarkable number of centromere features, including kinetochore assembly, chromosome segregation, and centromere clustering.

The variability in chromosome segregation accuracy of different neocentromeres has implications for our understanding of evolution and cancer. Some instances of speciation involve the formation of evolutionary new centromeres through centromere repositioning events that become fixed in the population [19,25]. A significant fraction of randomly isolated neocentromeres likely have the properties necessary to become evolutionary new centromeres. Other neocentromeres have elevated levels of chromosome segregation errors that could produce aneuploid progeny, which frequently are unfit [52] and yet sometimes promote survival under specific stress conditions, particularly in mitotic or somatic cells [53,54,55]. Approximately 3% of cancer cells have neochromosomes, many of which must have assembled neocentromeres [28]. Decreased chromosome segregation accuracy in neocentromere-containing cancer cells may promote the development of chemotherapy resistance. For example, aneuploidy gives rise to gene copy number variations that can confer resistance to chemotreatment in ovarian cancer [56]. On the other hand, very high levels of chromosome loss may decrease the survival of the cancer cells as extreme genome instability is associated with better prognosis in breast cancer patients [57]. Thus, in cancer cells, neocentromeres with lower levels of chromosome segregation accuracy might synergize with chemotherapy treatments to promote very high levels of genome instability and in turn, improve patient prognosis.

It is not yet clear what mechanisms determine the relative chromosome loss rates at different neocentromere positions in any organism or cell type. Native centromeres recruit error correction proteins [37,58], spindle assembly checkpoint proteins [59], and structural complexes such as condensin and monopolin [58,60] that are required for optimal chromosome segregation efficiency. We propose that the differences in chromosome segregation accuracy at different active neocentromeres may be due to the differential ability of neocentromeres to recruit these proteins or complexes. Whether this is due to the underlying DNA sequence and its transcriptional state, or to the epigenetic recruitment of other factors remains to be explored.

## Materials and Methods

### Strain construction for *CEN5* deletion

*CEN5* was deleted as previously described [32]. Lithium acetate transformation of PCR products with at least 70 bp of homology to the targeted gene was used for strain construction. Briefly, strains to be transformed were inoculated in liquid YPAD (10g/L yeast extract, 20g/L bactopeptone, 0.04g/L adenine, 0.08g/L uridine, 20g/L dextrose) and grown at 30°C for 16–18 h. Cultures were then diluted 1:166 in YPAD and grown at 30°C for 3–4 h. Cells were washed with water, then TELiAc (10mM Tris pH 7.5, 1mM EDTA, 100mM LiAc) and incubated in TELiAc with transformation DNA and 50μg sheared salmon sperm DNA (Ambion) for 30 min. 4 volumes PLATE mix (40% PEG, 10mM Tris pH 7.5, 1mM EDTA, 100mM LiAc) was then added and the transformation mix was incubated for 16–18 h at 20–24°C. Transformations were heat shocked at 42°C for 1 h, then plated on selective media with the exception of NAT1 marker transformations, which were recovered on non-selective media for 6 h prior to replica plating to selective media containing 400 μg/ml nourseothricin (Werner BioAgents). Strains were checked by PCR of genomic DNA.

### Chromatin Immunoprecipitation (ChIP)

ChIP was performed essentially as described in [32]. ChIP was performed using rabbit anti-Cse4 (CaCENP-A) antibodies [32], rabbit anti-histone H4 antibodies [61], and rabbit anti-histone H4K16Ac antibodies (Abcam). DNA pull-down efficiency was measured by qPCR using the Universal Probe Library (Roche Applied Science) with a LightCycler 480 PCR machine (Roche Applied Science) according to the manufacturer’s instructions. Enrichment was calculated as relative quantification of (+Ab/Input)-(-Ab/Input) using the second-derivative maximum to determine C^T^ values and corrections for primer efficiency values with the LightCycler 480 software (Roche Applied Science). For H4K16 ChIP, the H4K16 ChIP was normalized to ChIP of total H4.

### Array hybridization

Custom microarrays (Agilent SurePrint 8x60k) were designed with 60bp probes targeted towards all centromere sequences and the complete chromosome sequences of Chr4, Chr5 and Chr7. Labeling of ChIP DNA (input and anti-Cse4 IP) and hybridization of the arrays were performed according to the manufacturer’s instructions. Arrays were scanned with an Agilent SureScan scanner. Images were processed with the Agilent Feature Extraction software. The Log2 IP/WCE ratio data was normalized and plotted by chromosome position. Neocentromere positions were identified by areas of enrichment of Cse4 and were confirmed by qPCR.

### Reverse transcriptase qPCR

Strains were inoculated into YPAD and grown at 30°C for 16–18 hr. Cultures were then diluted 1:100 into YPAD and grown at 30°C for 4 hr. RNA was prepared using the MasterPure yeast RNA purification kit (Epicentre) according to the manufacturer’s instructions. RNA was treated with DNase (Epicentre) to remove contaminating genomic DNA. cDNA was prepared using the ProtoScript M-MuLV First Strand cDNA Synthesis Kit (New England Biolabs) according to the manufacturer’s instructions with oligo dT primers. cDNA was measured by qPCR using the Universal Probe Library (Roche Applied Science) with a LightCycler 480 PCR machine (Roche Applied Science) or Rotor-Gene SYBR Green master mix (Qiagen) with a Rotor-Gene cycler (Qiagen) according to the manufacturer’s instructions. Expression was calculated as the amount of cDNA from the gene of interest relative to the amount of *TEF1* cDNA in the same sample using the second-derivative maximum to determine C^T^ values and corrections for primer efficiency values.

### Fluctuation analysis of chromosome loss rates

Fluctuation analysis of loss rates was performed as described elsewhere [62] using the method of the median [63]. Briefly, strains were streaked for single colonies and grown on SDC-Uri for 2 days at 30°C. Per strain, 8 independent colonies were inoculated into 1ml liquid non-selective medium (YPAD) and grown overnight at 30°C with shaking. For heat stress assays, cultures were incubated at 39°C. For nicotinamide assays, colonies were inoculated in YPAD + 2mM nicotinamide and incubated at 30°C. For nocodazole assays, cells were inoculated in YPAD + 100μM nocodazole. Cultures were harvested by centrifugation and washed once in 1ml of sterile water. Dilutions were plated onto nonselective YPAD for total cell counts and selective media (SD+FOA for *URA3* loss) (Gold Biotechnology). Plates were incubated at 30°C for 2–3 days, and colony counts were used to calculate the rate of FOA^R^/cell division [62].

### SNP-RFLP

At least 8 individual colonies that lost *URA3* were isolated from 5-FOA plates after incubation in the fluctuation analysis. Colonies were streaked on YPAD plates and incubated at 30°C for 24 hr. Following genomic DNA extraction, PCR was performed on the right (5R) and left (5L) ends of Chr5 using primers as previously described [44]. Restriction digests were performed on resulting PCR products with Alu1 (5R) at 37°C and Taq1 (5L) at 60°C for approximately 16 hr. Restriction digests were run on 3% agarose gels to check for SNP homozygosity or heterozygosity (digested or non-digested alleles based on SNP present within PCR) [44].

### Hi-C data analyses

Hi-C experiments were performed as described previously using the Sau3AI restriction enzyme to digest the chromatin [64]. Sequencing was performed using 80bp paired-end reads. Reads were trimmed by 10bp from each end and remaining 60bps were mapped to *C*. *albicans* reference genome using BWA [65] with no mismatches allowed. Uniquely mapped read pairs were further binned into non-overlapping 10kb windows to create raw contact maps which were subsequently normalized using an iterative correction method [66]. The resulting normalized contact maps were used for heatmaps, virtual 4C plots and for prediction of centromere coordinates using Centurion algorithm [49]. Sequencing data for Hi-C libraries are available from the Short Read Archive accession number PRJNA308106.

### Genome analysis

*C*. *albicans* genome information was obtained from the *Candida* Genome Database at www.candidagenome.org. Assembly 21 was used for mapping neocentromere coordinates. Inverted repeats were identified with the Inverted Repeats Database (https://tandem.bu.edu/cgi-bin/irdb/irdb.exe), and tandem repeats were identified with the Tandem Repeats Database (https://tandem.bu.edu/cgi-bin/trdb/trdb.exe). GC% and GC skew ((G-C)/(G+C)) was calculated with FastPCR. Gene essentiality information for homologs of *C*. *albicans* genes was obtained from the *Saccharomyces* Genome Database at www.yeastgenome.org.

## Supporting Information

S1 Fig Schematic of centromere deletion transformants and positions. Proximal transformants were defined as those that exhibited reversible silencing of the *URA3* marker gene and that had the CENP-A binding region centered within 4kb of the deleted region. Transformants are both from this work and from Ketel *et al*. [32]. Distal neocentromere positions characterized immediately following transformation and those characterized following movement or additional analysis of more single colonies from the transformation are indicated on the bottom left.
(TIFF)

S2 Fig ChIP-chip identification of neocentromere positions. Native centromere and *cen5Δ* chromatin samples were immunoprecipitated with anti-CENP-A antibodies followed by hybridization to a tiling microarray. Ratios of IP samples to input (whole cell extract) are shown. All chromosome coordinates are on Chr5 and are indicated in kb. A. Native centromere. B. YJB11649 C. YJB12408 D. YJB12031 E. YJB12008 F. YJB10234 G. YJB12553 H. YJB12331 I. YJB10435 J. YJB9861 K. YJB11650 L. YJB12328 M. YJB9330 N. YJB12407 O. YJB9862
(TIFF)

S3 Fig Localization of neocentromere positions near 170kb. Anti-CENP-A ChIP analyzed by qPCR with primer pairs spaced approximately 500bp apart spanning the region from 163kb– 178kb on Chr5 for neocentromere strains YJB10779 (blue triangles) and YJB10780 (teal squares). Data shown are mean ± SEM of 2 technical replicates for qPCR. Position data for the neocentromere strains are representative of at least 3 independent biological replicates.
(TIFF)

S4 Fig Neocentromeres do not have consistent GC skew inversion patterns normally associated with evolutionarily constitutive origin sequences. 20kb of the DNA sequences surrounding the center chromosomal coordinate of the 8 native centromeres (A), the 20 neocentromeres (B), and the 20 size-matched random controls from Chr5 (C) were obtained from the Candida Genome Database and GC skew (G-C)/(G+C) was calculated over a 1500bp window. The center coordinate of the 20kb window and 2.5kb borders to each side of the center are marked with dashed lines.
(TIFF)

S5 Fig Association of *C*. *albicans* centromeres and neocentromeres with repeat sequences. A. Schematic of native centromeres in *C*. *albicans* and associated repeats. Centromere regions as annotated in the Candida Genome Database are indicated in pink. Tandem and inverted repeats are in shades of blue with degree of homology indicated on the blue color bar scale (see scale). Long terminal repeats (LTRs) are shown in green. The 3’ end of the *ALS2* gene adjacent to CEN6 containing many tandem repeats indicated in purple. Reproduced with permission from [60]. B. The distance between 20 random loci on Chr5 (grey circles) and the center point of each neocentromere strain (magenta circles) and the edge of the closest repeat element is shown in basepairs. There were no significant differences between these two groups (t-test, p>0.05). Native centromeres (purple circles) are shown for comparison.
(TIFF)

S6 Fig Transcriptional activity is repressed following neocentromere formation. Homozygous neocentromere strains YJB10777 (4.5kb center), YJB10779 (173.5kb center), YJB10780 (166kb center), YJB12026 (900kb center), YJB12027 (800kb center), YJB12028 (72.5kb center), and JYB12330 (826.5kb center) were grown in YPAD for 4 h. mRNA levels for (A) *ORF19*.*6670*, (B) *ORF19*.*1122*, (C) *ORF19*.*575*, (D) *ORF19*.*576*, (E) *ORF19*.*1285*, (F) *ORF19*.*1283*, (G) *ORF19*.*5693* and (H) *ORF19*.*3347* relative to the reference gene *TEF1* were measured by qRT-PCR. Data shown are mean ± SEM of 3 biological replicates. * p<0.05 by ANOVA and Tukey post-tests.
(TIFF)

S7 Fig Neocentromere chromosome loss rate is not dependent on length or repeat elements. A. The fold-difference in *URA3* loss rate between the mean rate for the native centromere strain and the mean rate of each neocentromere strain was plotted as a function of the length of the neocentromere CENP-A binding region. Correlation between these two variables was very low (r^2^ = 0.0001). B. The fold-difference in *URA3* loss rate between the mean rate for the native centromere strain and the mean rate of each neocentromere strain was plotted as a function of the distance between the neocentromere position to the closest repeat element. Correlation between these two variables was very low (r^2^ = 0.00003). C. The fold-difference in *URA3* loss rate between the mean rate for the native centromere strain and the mean rate of each neocentromere strain was plotted as a function of the fraction of the neocentromere CENP-A bound region that includes ORFs. Correlation between these two variables was low to moderate (r^2^ = 0.23).
(TIFF)

S8 Fig Alteration of acetylation of histone H4K16 increases chromosome loss rates. A. Anti-H4K16Ac ChIP and anti-H4 ChIP samples were analyzed by qPCR with primers pairs spaced approximately 1kb apart spanning the region from 464 – 476kb on Chr5 RM1000 strain YJB7617 in YPAD (magenta diamonds) and YPAD with 2mM nicotinamide (NAM) (purple squares). H4K16Ac ChIP was normalized to total H4 levels by anti-H4 ChIP. Data shown are mean ± SEM of 2 technical replicates for qPCR and are representative of at least 3 independent biological replicates. B. Cultures of each strain were grown in YPAD for 24 h at 30°C with no drug treatment (magenta) or treatment with 2mM nicotinamide (purple). Loss of *URA3* was quantified by plating cells on non-selective media and on media containing 5-FOA to select for loss of *URA3*. Colony counts were used to calculate the rate of loss per cell division. Results are the mean ± SEM of the rates calculated from at least 3 experiments, each with 8 cultures per condition. p<0.01 for nicotinamide treatment differences and p>0.05 for nicotinamide*strain interaction by two-way ANOVA.
(TIFF)

S9 Fig Strains with native centromeres and neocentromeres have an increase in *URA3* loss rates with nocodazole treatment. Fluctuation analysis of loss of *URA3* in control (*INT1/int1Δ*::*ura3*) and neocentromere *(CEN5/cen5Δ*::*ura3*) strains. Cultures of each strain were grown in YPAD for 24 h at 30°C with no drug treatment (magenta) or treatment with 100μM nocodazole (purple). Loss of *URA3* was quantified by plating cells on non-selective media and on media containing 5-FOA to select for loss of *URA3*. Colony counts were used to calculate the rate of loss per cell division. Results are the mean ± SEM of the rates calculated from at least 3 experiments, each with 8 cultures per condition. p<0.05 for differences between control and nocodazole treatments by ANOVA.
(TIFF)

S10 Fig Centromere clustering occurs between chromosomes in *C*. *albicans*. Red lines mark centromeres. Green lines indicate neocentromere positions. Black diamond indicates the viewpoint for the plotted interaction profiles. A. Virtual 4C plots from the 10kb sequence surrounding the center of native *CEN1* showing log-scaled Hi-C contact counts for all *C*. *albicans* chromosomes in the wild type strain. B. Virtual 4C plots from the 10kb sequence surrounding the center of native *CEN7* showing log-scaled Hi-C contact counts for all *C*. *albicans* chromosomes in the wild type strain.
(TIFF)

S11 Fig Centromere clustering depends on functional kinetochore assembly and is lost at the former native centromere region following neocentromere formation. Red lines mark centromeres. Green lines indicate neocentromere positions. Black diamond indicates the viewpoint for the plotted interaction profiles. A. Virtual 4C plots from the 10kb sequence surrounding the center of native *CEN5* showing log-scaled Hi-C contact counts for all *C*. *albicans* chromosomes in the YJB10777 (4.5kb neocentromere, at 0.0045Mb in diagram) strain. B. Virtual 4C plots from the 10kb sequence surrounding the center of native *CEN5* showing log-scaled Hi-C contact counts for all *C*. *albicans* chromosomes in the YJB10780 (166kb neocentromere, at 0.166Mb in diagram) strain.
(TIFF)

S1 Table Neocentromere genomic features. Neocentromere positions and sizes based on ChIP-chip experiments with ORFs and ORF overlap identified using the Candida Genome Database. Essential genes were identified using the Yeast Genome Database. Transcription levels in YPD from Bruno *et al*. 2010 are indicated in a log2 scale of reads per kilobase per million mapped reads (RPKM) and fold-change relative to the median transcription level for all genes based on mean transcriptional activity in two YPD replicates with lighter shading indicating lower transcription levels and darker shading indicating higher transcription levels.
(PDF)

S2 Table SNP-RFLP analysis of homozygous (whole chromosome loss) and heterozygous (recombination-based loss) FOA^R^ isolates from fluctuation analysis. The number of isolates with homozygous SNP markers at both ends of Chr5 are indicated along with the total number of FOA^R^ isolates tested.
(PDF)

S3 Table Centromere and neocentromere calls with Centurion algorithm. (PDF)
