# Supplementary figures and images for: Neocentromeres Provide Chromosome Segregation Accuracy and Centromere Clustering to Multiple Loci along a Candida albicans Chromosome
Source: PLoS Genet. 2016 Sep 23;12(9):e1006317. doi: 10.1371/journal.pgen.1006317 (PMC5035033; doi:10.1371/journal.pgen.1006317)

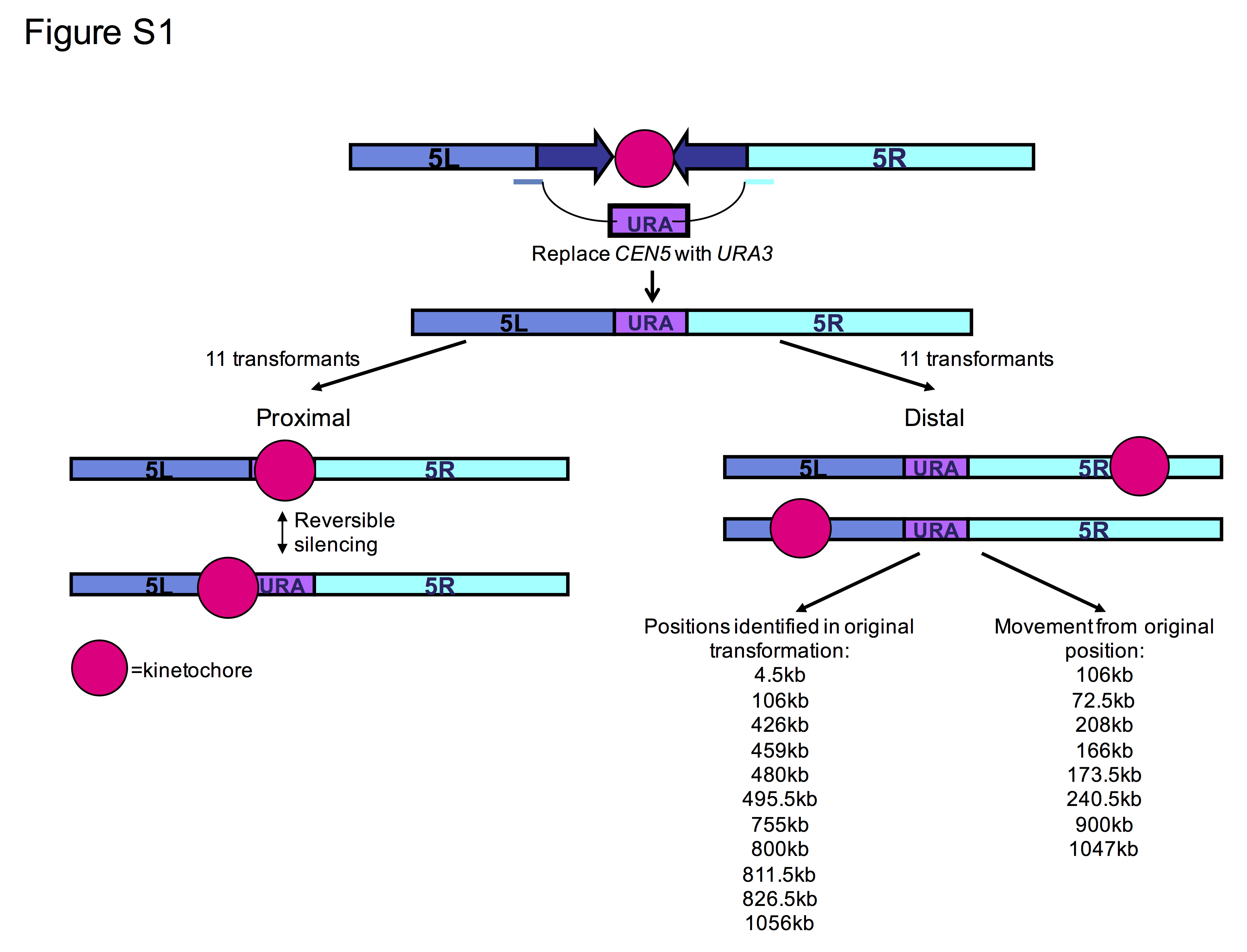

Supplement: S1 Fig — Proximal transformants were defined as those that exhibited reversible silencing of the URA3 marker gene and that had the CENP-A binding region centered within 4kb of the deleted region. Transformants are both from this work and from Ketel et al. [32]. Distal neocentromere positions characterized immediately following transformation and those characterized following movement or additional analysis of more single colonies from the transformation are indicated on the bottom left. (TIFF) [file pgen.1006317.s001.tiff]

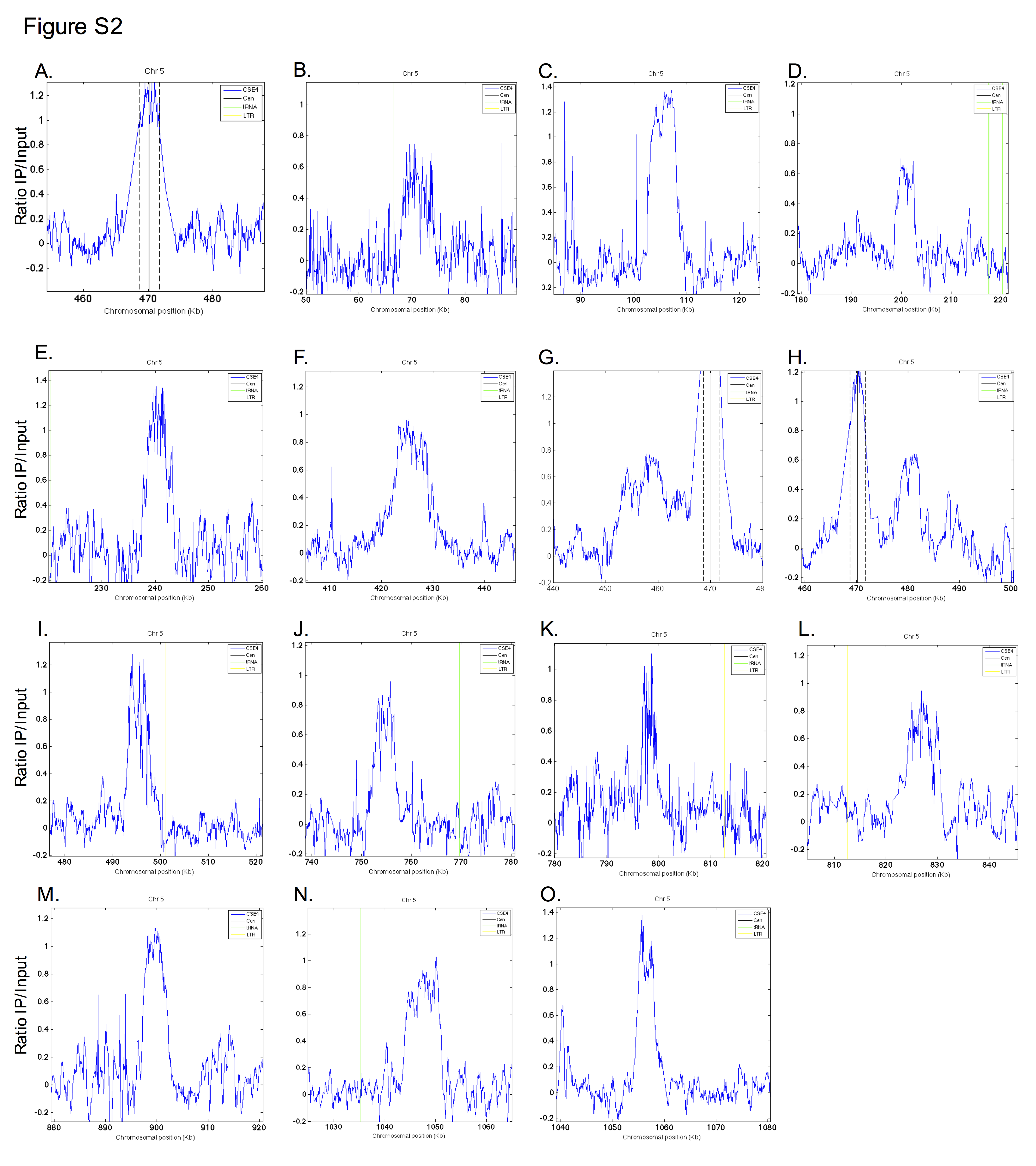

Supplement: S2 Fig — Native centromere and cen5Δ chromatin samples were immunoprecipitated with anti-CENP-A antibodies followed by hybridization to a tiling microarray. Ratios of IP samples to input (whole cell extract) are shown. All chromosome coordinates are on Chr5 and are indicated in kb. A. Native centromere. B. YJB11649 C. YJB12408 D. YJB12031 E. YJB12008 F. YJB10234 G. YJB12553 H. YJB12331 I. YJB10435 J. YJB9861 K. YJB11650 L. YJB12328 M. YJB9330 N. YJB12407 O. YJB9862 (TIFF) [file pgen.1006317.s002.tiff]

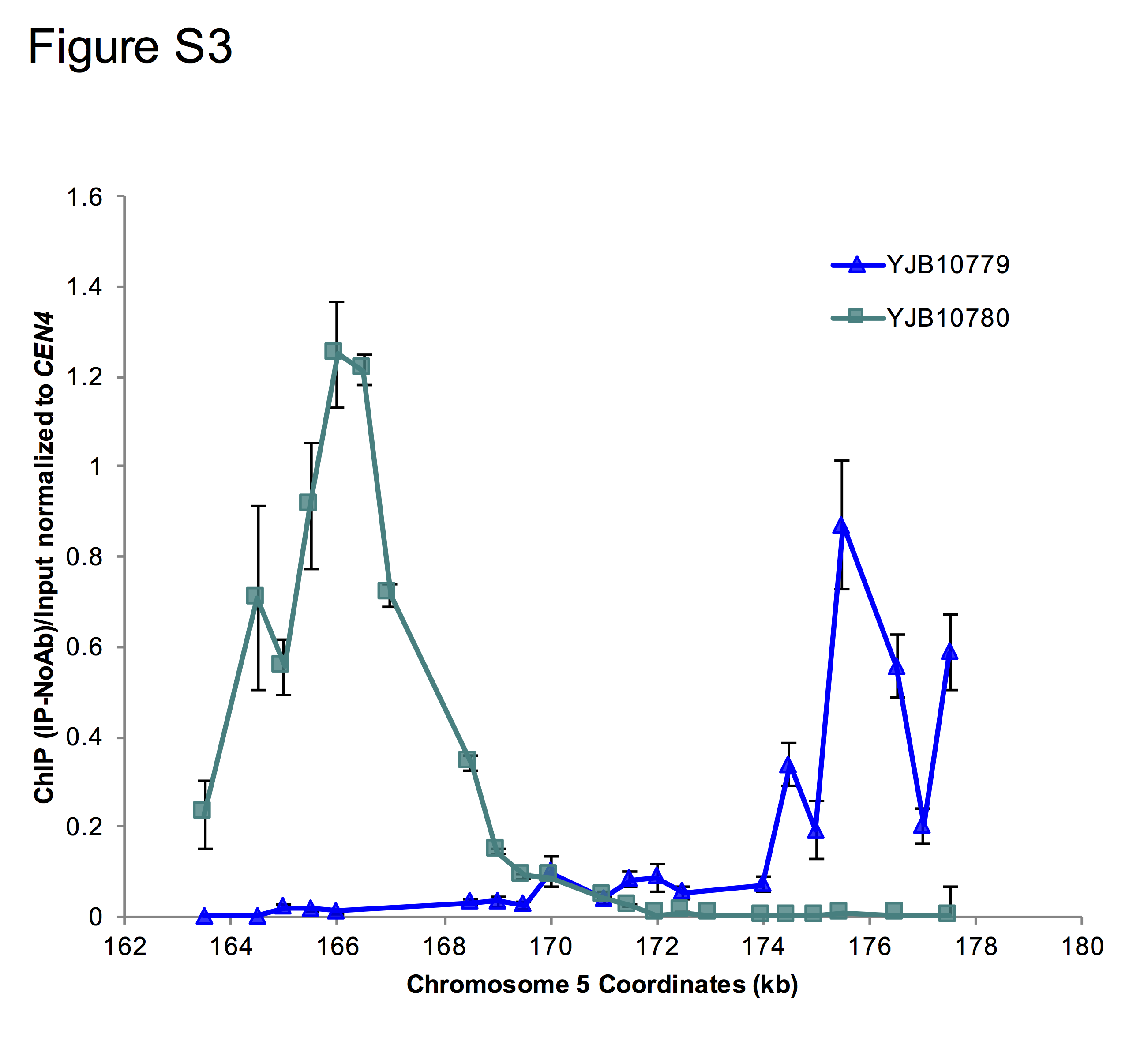

Supplement: S3 Fig — Anti-CENP-A ChIP analyzed by qPCR with primer pairs spaced approximately 500bp apart spanning the region from 163kb– 178kb on Chr5 for neocentromere strains YJB10779 (blue triangles) and YJB10780 (teal squares). Data shown are mean ± SEM of 2 technical replicates for qPCR. Position data for the neocentromere strains are representative of at least 3 independent biological replicates. (TIFF) [file pgen.1006317.s003.tiff]

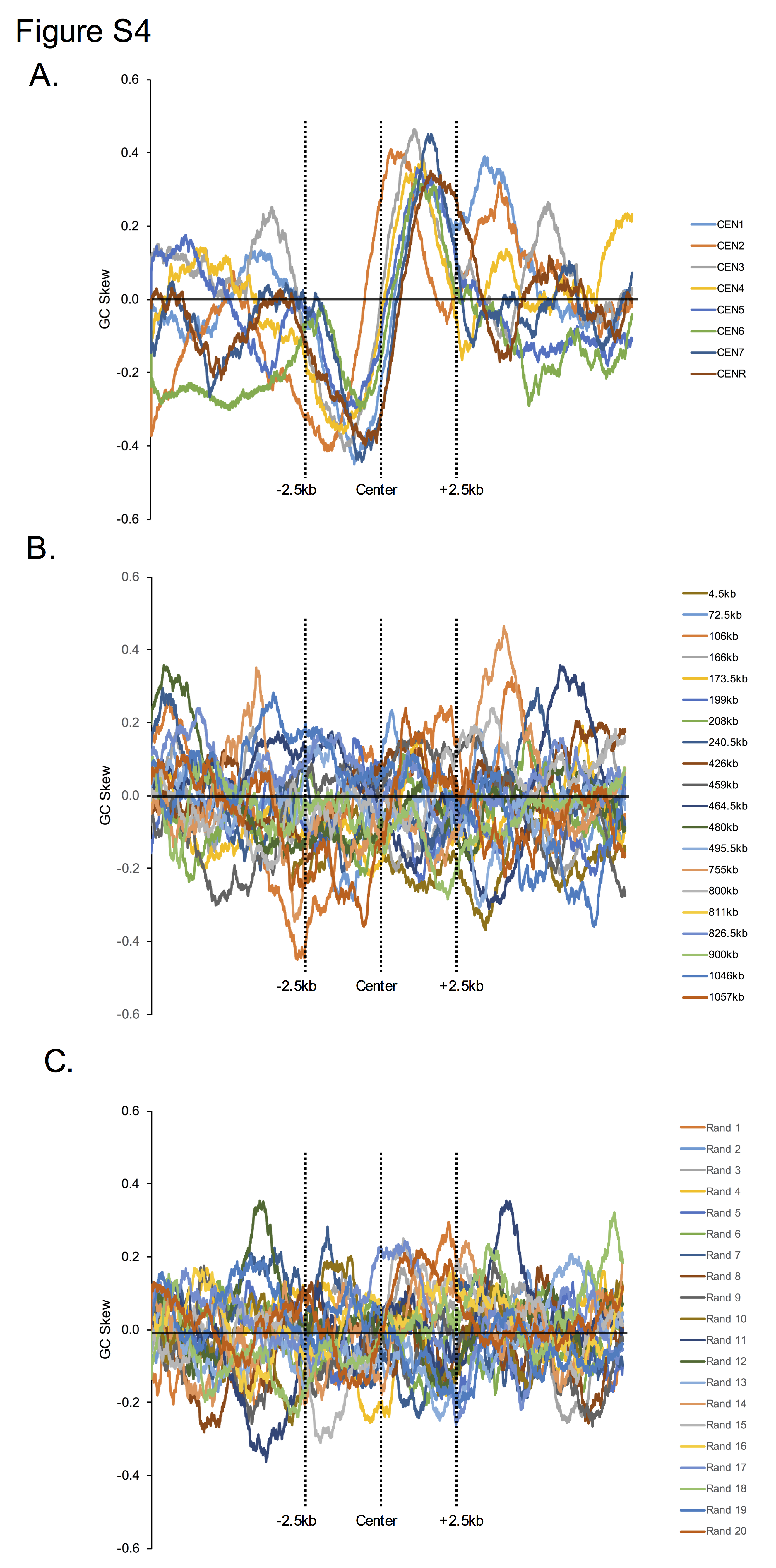

Supplement: S4 Fig — 20kb of the DNA sequences surrounding the center chromosomal coordinate of the 8 native centromeres (A), the 20 neocentromeres (B), and the 20 size-matched random controls from Chr5 (C) were obtained from the Candida Genome Database and GC skew (G-C)/(G+C) was calculated over a 1500bp window. The center coordinate of the 20kb window and 2.5kb borders to each side of the center are marked with dashed lines. (TIFF) [file pgen.1006317.s004.tiff]

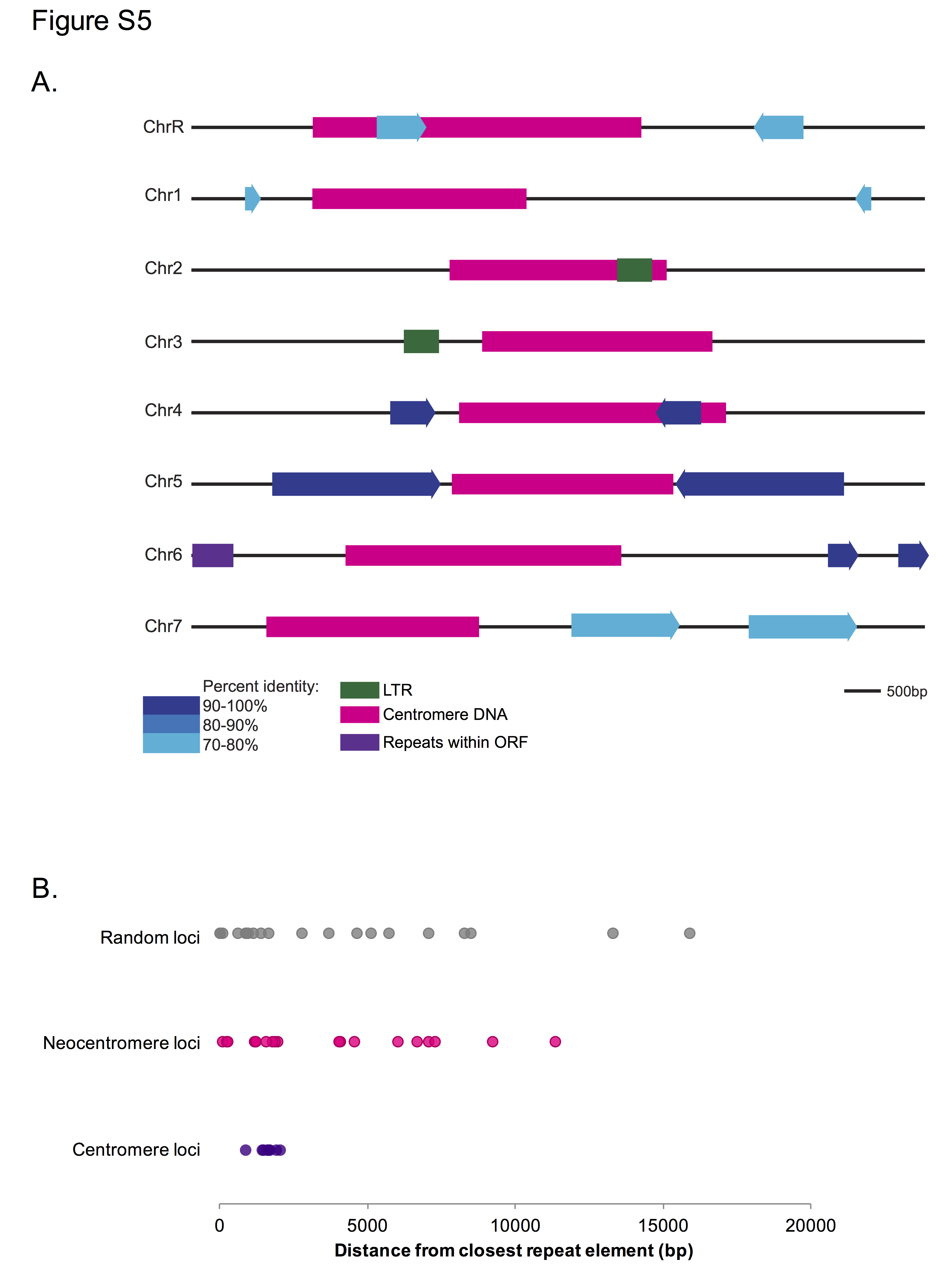

Supplement: S5 Fig — A. Schematic of native centromeres in C. albicans and associated repeats. Centromere regions as annotated in the Candida Genome Database are indicated in pink. Tandem and inverted repeats are in shades of blue with degree of homology indicated on the blue color bar scale (see scale). Long terminal repeats (LTRs) are shown in green. The 3’ end of the ALS2 gene adjacent to CEN6 containing many tandem repeats indicated in purple. Reproduced with permission from [60]. B. The distance between 20 random loci on Chr5 (grey circles) and the center point of each neocentromere strain (magenta circles) and the edge of the closest repeat element is shown in basepairs. There were no significant differences between these two groups (t-test, p>0.05). Native centromeres (purple circles) are shown for comparison. (TIFF) [file pgen.1006317.s005.tiff]

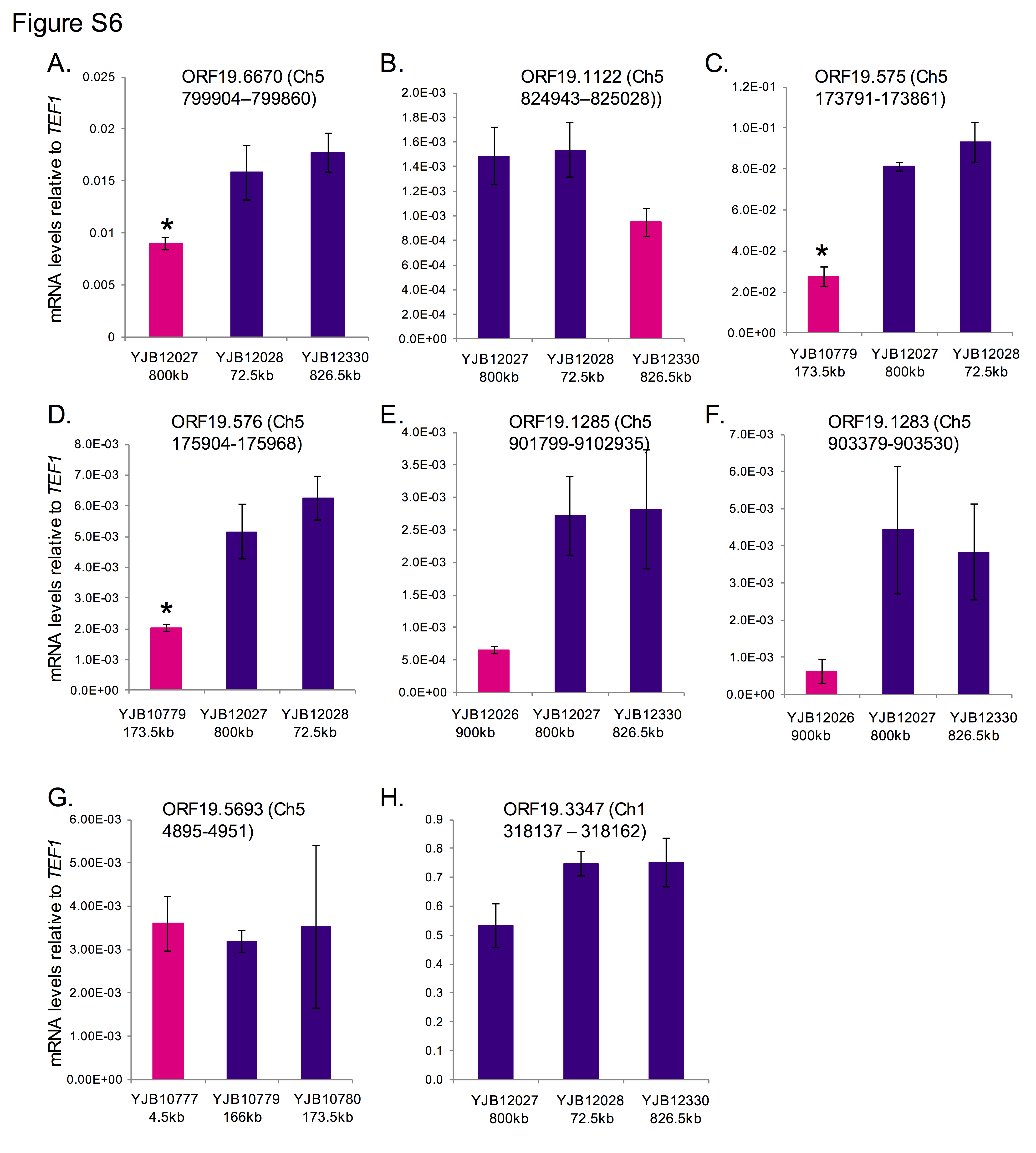

Supplement: S6 Fig — Homozygous neocentromere strains YJB10777 (4.5kb center), YJB10779 (173.5kb center), YJB10780 (166kb center), YJB12026 (900kb center), YJB12027 (800kb center), YJB12028 (72.5kb center), and JYB12330 (826.5kb center) were grown in YPAD for 4 h. mRNA levels for (A) ORF19.6670, (B) ORF19.1122, (C) ORF19.575, (D) ORF19.576, (E) ORF19.1285, (F) ORF19.1283, (G) ORF19.5693 and (H) ORF19.3347 relative to the reference gene TEF1 were measured by qRT-PCR. Data shown are mean ± SEM of 3 biological replicates. * p<0.05 by ANOVA and Tukey post-tests. (TIFF) [file pgen.1006317.s006.tiff]

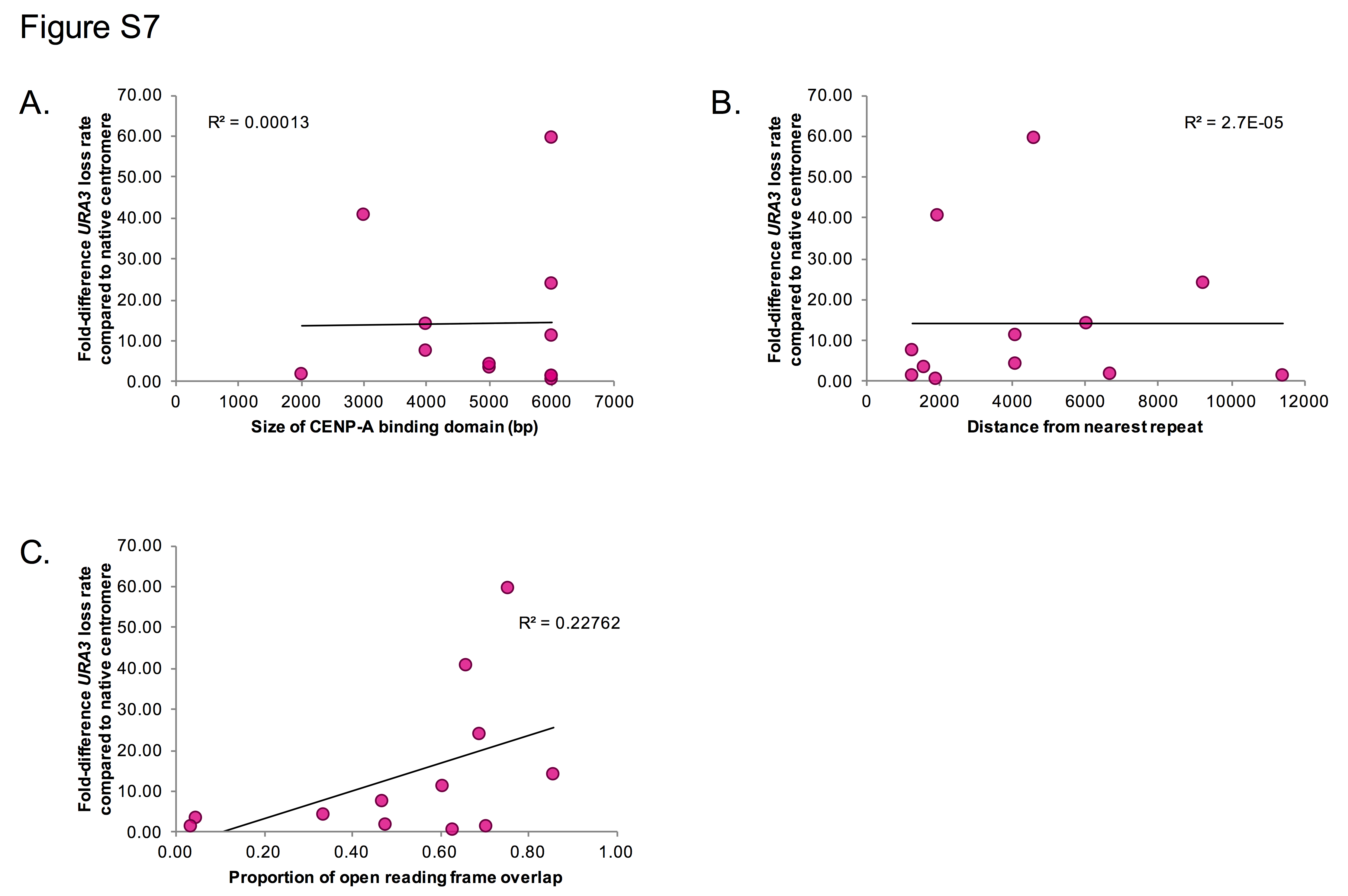

Supplement: S7 Fig — A. The fold-difference in URA3 loss rate between the mean rate for the native centromere strain and the mean rate of each neocentromere strain was plotted as a function of the length of the neocentromere CENP-A binding region. Correlation between these two variables was very low (r2 = 0.0001). B. The fold-difference in URA3 loss rate between the mean rate for the native centromere strain and the mean rate of each neocentromere strain was plotted as a function of the distance between the neocentromere position to the closest repeat element. Correlation between these two variables was very low (r2 = 0.00003). C. The fold-difference in URA3 loss rate between the mean rate for the native centromere strain and the mean rate of each neocentromere strain was plotted as a function of the fraction of the neocentromere CENP-A bound region that includes ORFs. Correlation between these two variables was low to moderate (r2 = 0.23). (TIFF) [file pgen.1006317.s007.tiff]

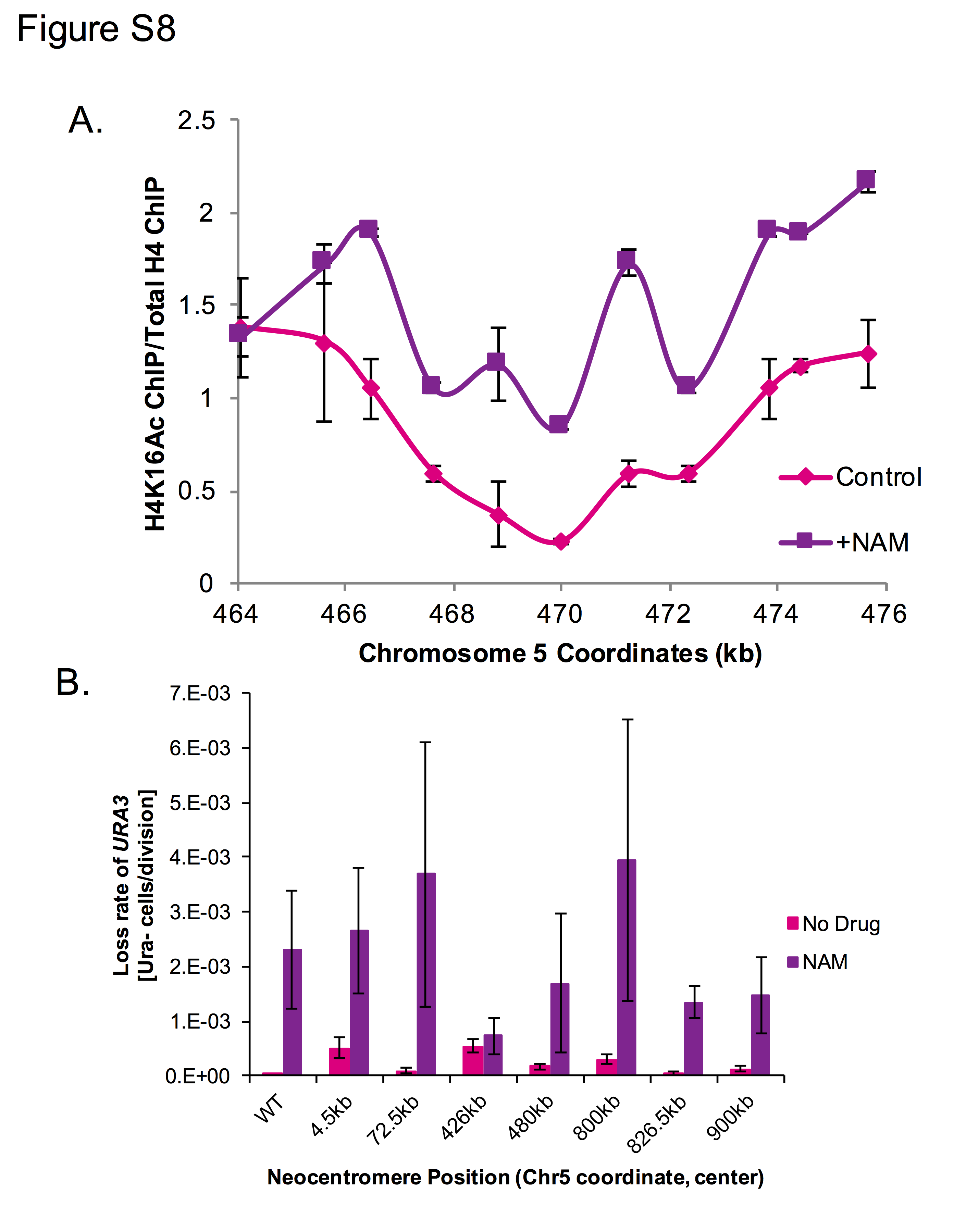

Supplement: S8 Fig — A. Anti-H4K16Ac ChIP and anti-H4 ChIP samples were analyzed by qPCR with primers pairs spaced approximately 1kb apart spanning the region from 464 – 476kb on Chr5 RM1000 strain YJB7617 in YPAD (magenta diamonds) and YPAD with 2mM nicotinamide (NAM) (purple squares). H4K16Ac ChIP was normalized to total H4 levels by anti-H4 ChIP. Data shown are mean ± SEM of 2 technical replicates for qPCR and are representative of at least 3 independent biological replicates. B. Cultures of each strain were grown in YPAD for 24 h at 30°C with no drug treatment (magenta) or treatment with 2mM nicotinamide (purple). Loss of URA3 was quantified by plating cells on non-selective media and on media containing 5-FOA to select for loss of URA3. Colony counts were used to calculate the rate of loss per cell division. Results are the mean ± SEM of the rates calculated from at least 3 experiments, each with 8 cultures per condition. p<0.01 for nicotinamide treatment differences and p>0.05 for nicotinamide*strain interaction by two-way ANOVA. (TIFF) [file pgen.1006317.s008.tiff]

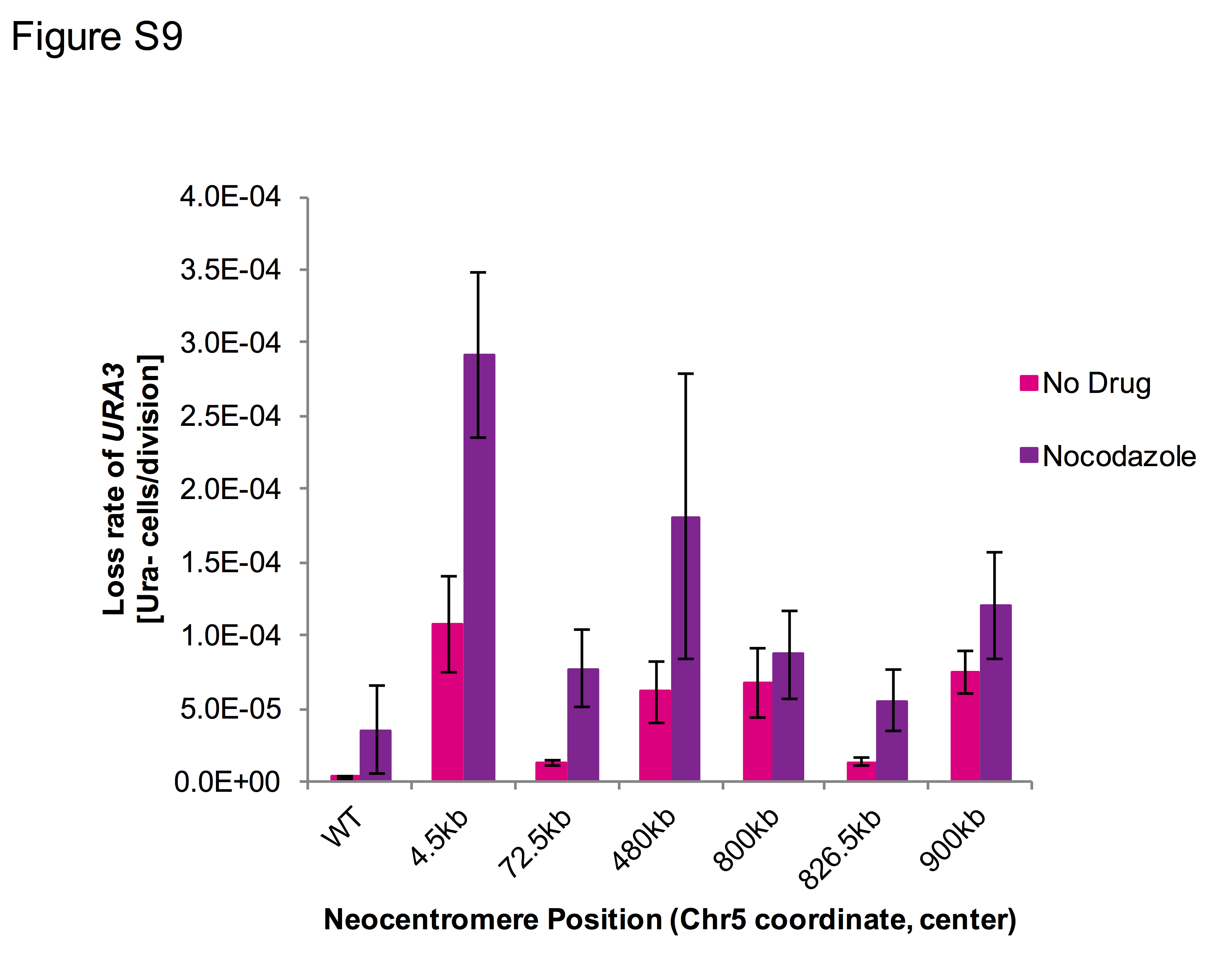

Supplement: S9 Fig — Fluctuation analysis of loss of URA3 in control (INT1/int1Δ::ura3) and neocentromere (CEN5/cen5Δ::ura3) strains. Cultures of each strain were grown in YPAD for 24 h at 30°C with no drug treatment (magenta) or treatment with 100μM nocodazole (purple). Loss of URA3 was quantified by plating cells on non-selective media and on media containing 5-FOA to select for loss of URA3. Colony counts were used to calculate the rate of loss per cell division. Results are the mean ± SEM of the rates calculated from at least 3 experiments, each with 8 cultures per condition. p<0.05 for differences between control and nocodazole treatments by ANOVA. (TIFF) [file pgen.1006317.s009.tiff]

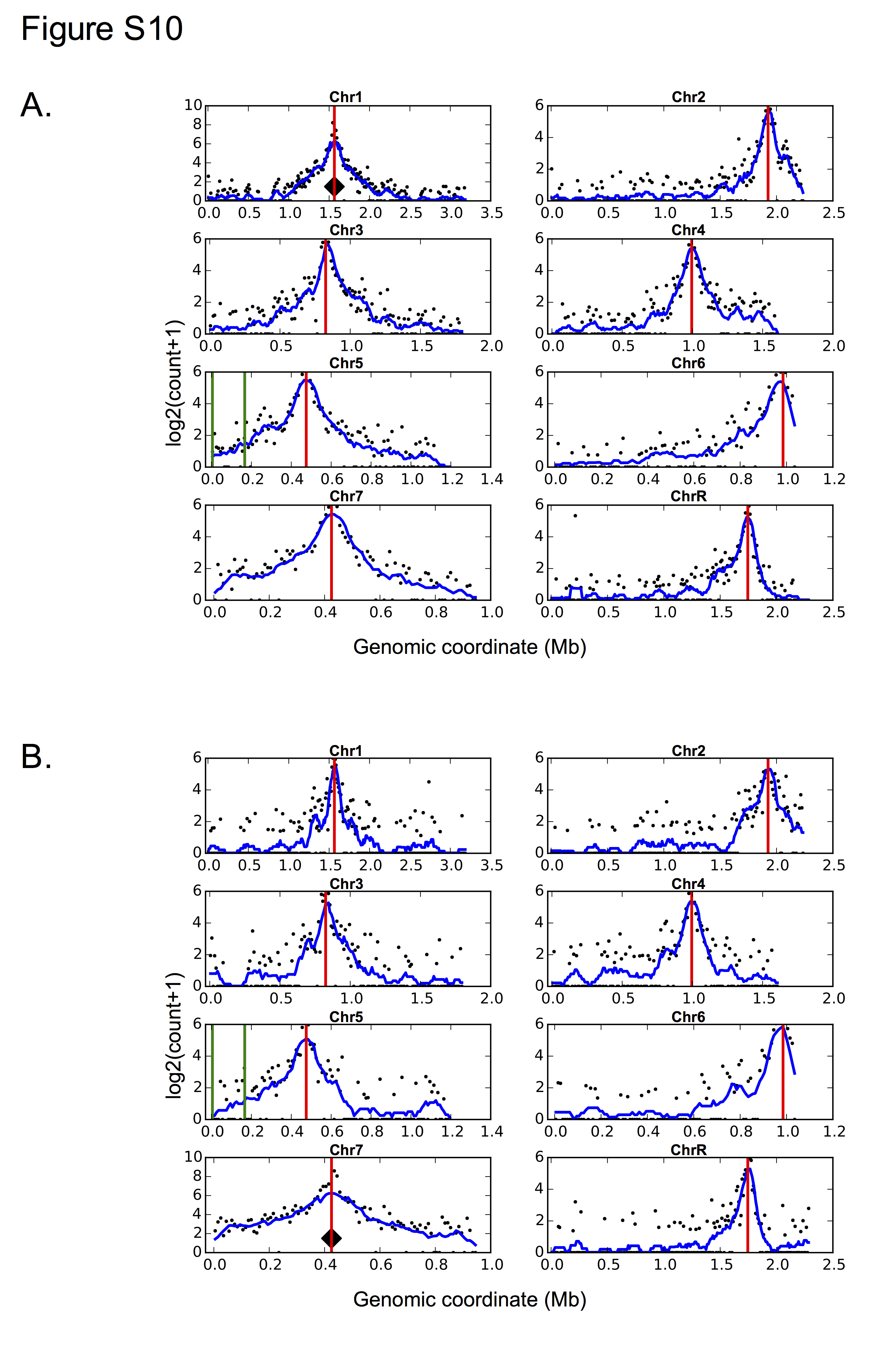

Supplement: S10 Fig — Red lines mark centromeres. Green lines indicate neocentromere positions. Black diamond indicates the viewpoint for the plotted interaction profiles. A. Virtual 4C plots from the 10kb sequence surrounding the center of native CEN1 showing log-scaled Hi-C contact counts for all C. albicans chromosomes in the wild type strain. B. Virtual 4C plots from the 10kb sequence surrounding the center of native CEN7 showing log-scaled Hi-C contact counts for all C. albicans chromosomes in the wild type strain. (TIFF) [file pgen.1006317.s010.tiff]

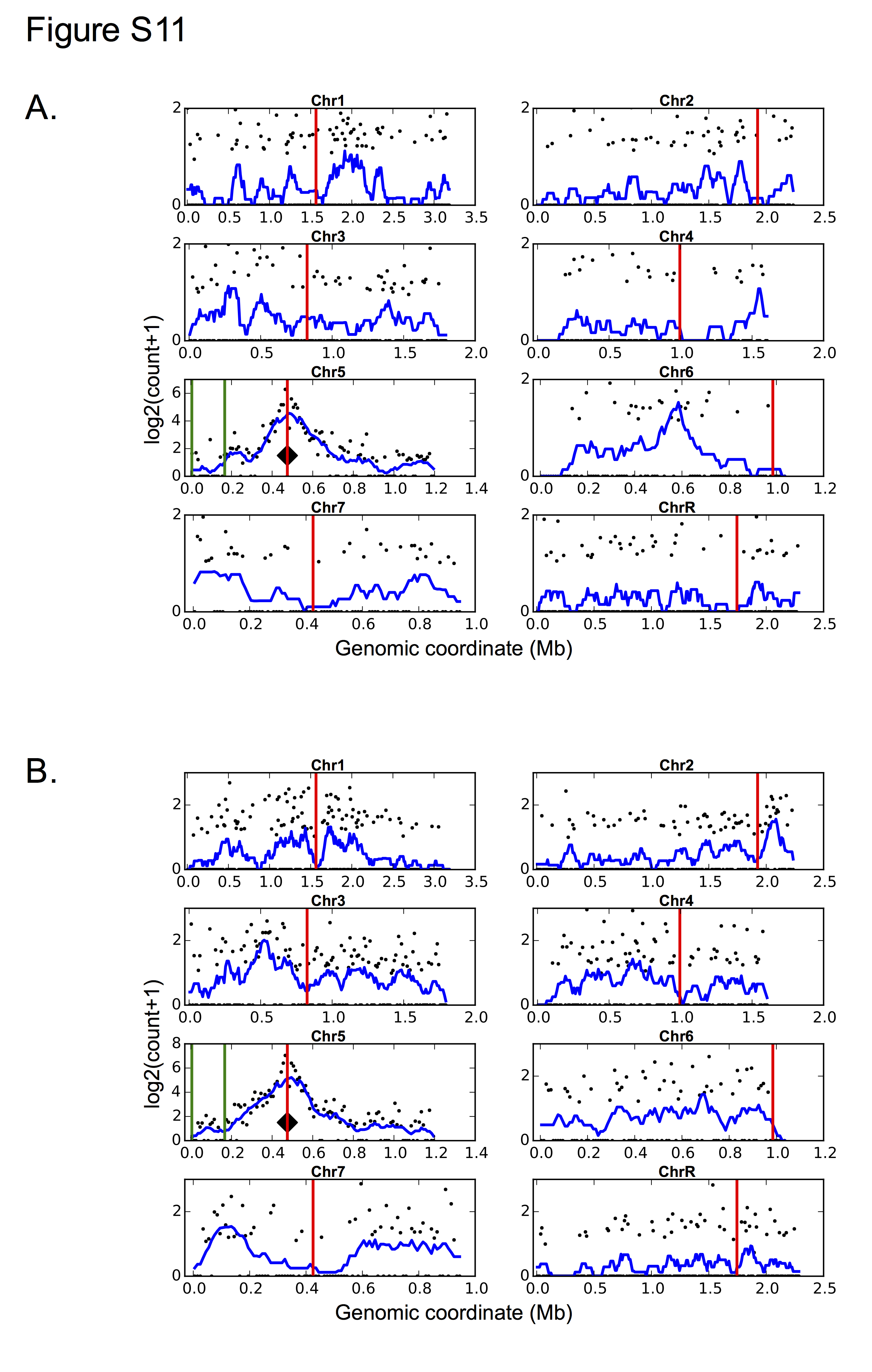

Supplement: S11 Fig — Red lines mark centromeres. Green lines indicate neocentromere positions. Black diamond indicates the viewpoint for the plotted interaction profiles. A. Virtual 4C plots from the 10kb sequence surrounding the center of native CEN5 showing log-scaled Hi-C contact counts for all C. albicans chromosomes in the YJB10777 (4.5kb neocentromere, at 0.0045Mb in diagram) strain. B. Virtual 4C plots from the 10kb sequence surrounding the center of native CEN5 showing log-scaled Hi-C contact counts for all C. albicans chromosomes in the YJB10780 (166kb neocentromere, at 0.166Mb in diagram) strain. (TIFF) [file pgen.1006317.s011.tiff]
