# Supplementary material for: Neocentromeres Provide Chromosome Segregation Accuracy and Centromere Clustering to Multiple Loci along a Candida albicans Chromosome
Source: PLoS Genet. 2016 Sep 23;12(9):e1006317. doi: 10.1371/journal.pgen.1006317 (PMC5035033; doi:10.1371/journal.pgen.1006317)
Supplement: S1 Table — Neocentromere positions and sizes based on ChIP-chip experiments with ORFs and ORF overlap identified using the Candida Genome Database. Essential genes were identified using the Yeast Genome Database. Transcription levels in YPD from Bruno et al. 2010 are indicated in a log2 scale of reads per kilobase per million mapped reads (RPKM) and fold-change relative to the median transcription level for all genes based on mean transcriptional activity in two YPD replicates with lighter shading indicating lower transcription levels and darker shading indicating higher transcription levels. (PDF) [file pgen.1006317.s012.pdf]

Supplementary Table S1: Neocentromere genomic features. Neocentromere positions and sizes based on ChIP-chip experiments with ORFs and ORF overlap identified using the Candida Genome Database. Essential genes were identified using the Yeast Genome Database. Transcription levels in YPD from Bruno *et al.* 2010 in are indicated in a log(2) scale of reads per kilobase per million mapped reads (RPKM) and fold-change relative to the median transcription level for all genes based on mean transcriptional activity in two YPD replicates with lighter shading indicating lower transcription levels and darker shading indicating higher transcription levels.

Neocentromere position center coordinate (Chr5) (bp)	Estimated CENP-A binding coordinates (Chr5) (bp)	CENP-A binding domain size (bp)	ORFs within CENP-A region (Bold means essential gene in *S. cerevisiae*)	Overlap with CENP-A (Bold means entire ORF overlaps)	Transcription in YPD (log2 RPKM)	Transcription in YPD (Fold-change relative to median transcript level in YPD)	Strain Number(s) of Isolates		Strain Number of Original Transformant	Reference
							Heterozygous *CEN5* deletion	Homozygous *CEN5* deletion		
4500	3000-6000	3000	orf19.5698	**810**	6.99	5.1	YJB9907	YJB10777	YJB9907	(Ketel *et al.*, 2009)
			orf19.5693	1,170 (3')	6.13	2.8				
72500	70500-74500	4000	orf19.952	904 (3')	4.12	0.7	YJB11650	YJB12028	YJB11649	This work.
			orf19.951	**489**	8.33	12.8				
			orf19.949	482 (3')	4.90	1.2				
106000	103000-109000	6000	orf19.938	**1446**	-1.51	0.01	YJB12408	YJB13284	YJB9907, YJB12408	This work.
			orf19.936	**2325**	-4.60	0.002				
166000	163500-168500	5000	orf19.570	**2145**	-0.15	0.04	N/A	YJB10780	YJB9929	(Ketel *et al.*, 2009)
173500	171000-176000	5000	orf19.575	**3750**	3.97	0.6	N/A	YJB10779	YJB9929	(Ketel *et al.*, 2009)
			orf19.576	441 (5')	4.96	1.2				
199000	196000-202000	6000	orf19.586	621 (5')	6.19	2.9	YJB12031	YJB12021	YJB9929	This work.
			orf19.587	**1194**	4.82	1.1				
			orf19.588	**319**	4.76	1.1				
			orf19.1981	**489**	5.22	1.5				
			orf19.1980	**1599**	-0.71	0.02				
208000	207000-209000	2000	orf19.1978	953 (5')	2.87	0.3	YJB12402	YJB12015	YJB9929	(Ketel *et al.*, 2009)
240500	237500-243500	6000	None	N/A	N/A	N/A	YJB12008	YJB12017	YJB9929	This work.
426000	423000-429000	6000	orf19.3187	724 (5')	4.31	0.8	YJB10234	N/A	YJB10234	This work.
			orf19.3185	**2349**	7.08	5.4				
			orf19.3184	**648**	4.10	0.7				
			orf19.3183	**675**	6.07	2.7				
			orf19.3181.1	116 (3')	5.69	2.1				
459000	457000-461000	4000	orf19.3167	857 (3')	6.26	3.1	YJB12553	N/A	YJB12533	This work. (Thakur and Sanyal, 2013)
			**orf19.3166**	2807 (5')	4.26	0.8				

464500 (Proximal)	463000-466000	3000	orf19.3163	**987**	3.76	0.5	11 isolates (Ketel *et al.* strains: YJB9909, YJB9915, YJB9916, YJB9926)	YJB12549	11 isolates (Ketel *et al.* strains: YJB9909, YJB9915, YJB9916, YJB9926)	This work. (Ketel *et al.*, 2009)
			**orf19.3161**	**963**	6.40	3.4				
			NOVEL-Ca21chr5-019	116 (3')	4.31	0.8				
480000	478000-482000	4000	**orf19.4221**	**1695**	4.60	1.0	YJB12331	N/A	YJB12331	This work. (Thakur and Sanyal, 2013)
			orf19.4222	1730 (5')	2.99	0.3				
495500	493000-498000	5000	**orf19.4230**	223 (3')	7.02	5.2	YJB10436	N/A	YJB10436	This work.
755000	753000-757000	4000	orf19.2647	**1587**	2.31	0.2	YJB9861	N/A	YJB9861	This work.
			orf19.2646	950 (3')	2.62	0.2				
800000	797000-803000	6000	orf19.6667	**507**	4.70	1.0	YJB11649	YJB12027	YJB11649	This work.
			orf19.6668	**510**	5.26	1.5				
			orf19.6670	**1383**	4.16	0.7				
			orf19.6671	**1737**	3.45	0.4				
811500	810000-813000	3000	orf19.6676	307 (3')	5.12	1.4	YJB9929	YJB12024	YJB9929	(Ketel *et al.*, 2009)
			orf19.6678	**1122**	2.66	0.3				
826500	824000-829000	5000	orf19.1122	**843**	-2.85	0.01	YJB12328	YJB12330	YJB12328	This work.
			orf19.1121	**822**	5.22	1.5				
900000	897000-903000	6000	orf19.1285	**2796**	3.04	0.3	YJB9930	YJB12018	YJB9929	This work.
			orf19.1283	832 (3')	3.07	0.3				
1047000	1044000-1050000	6000	orf19.3967	198 (3')	9.25	24.3	YJB12407	YJB12019	YJB9929	This work.
1056000	1054000-1058000	4000	orf19.3969	2014 (3')	2.65	0.3	YJB9862	N/A	YJB9862	This work.
