# Supplementary material for: Neocentromeres Provide Chromosome Segregation Accuracy and Centromere Clustering to Multiple Loci along a Candida albicans Chromosome
Source: PLoS Genet. 2016 Sep 23;12(9):e1006317. doi: 10.1371/journal.pgen.1006317 (PMC5035033; doi:10.1371/journal.pgen.1006317)
Supplement: S2 Table — The number of isolates with homozygous SNP markers at both ends of Chr5 are indicated along with the total number of FOAR isolates tested. (PDF) [file pgen.1006317.s013.pdf]

Supplementary Table S2: SNP-RFLP analysis of homozygous (whole chromosome loss) and heterozygous (recombination-based loss) FOA^R isolates from fluctuation analysis. The number of isolates with homozygous SNP markers at both ends of Chr5 are indicated along with the total number of FOA^R isolates tested.

Centromere/ Neocentromere Position	Number Chromosome Loss/Number FOA^R Isolates Tested (%)			
	30°C/No Drug	39°C/No Drug	30°C/+NAM	30°C/+Nocodazole
Native centromere	6/19 (32%)	3/8 (38%)	5/8 (63%)	7/8 (88%)
4.5kb	12/15 (80%)	6/8 (75%)	5/8 (63%)	8/8 (100%)
72.5kb	14/16 (88%)	8/8 (100%)	6/8 (75%)	7/8 (88%)
426kb	14/16 (88%)	8/8 (100%)	8/8 (100%)	5/8 (63%)
480kb	7/16 (44%)	3/8 (38%)	3/8 (38%)	2/8 (25%)
800kb	11/16 (69%)	8/8 (100%)	7/8 (88%)	3/8 (38%)
826.5kb	11/15 (73%)	4/8 (50%)	8/8 (100%)	7/8 (88%)
900kb	11/16 (69%)	4/8 (50%)	6/10 (60%)	6/8 (75%)
