# Supplementary material for: Neocentromeres Provide Chromosome Segregation Accuracy and Centromere Clustering to Multiple Loci along a Candida albicans Chromosome
Source: PLoS Genet. 2016 Sep 23;12(9):e1006317. doi: 10.1371/journal.pgen.1006317 (PMC5035033; doi:10.1371/journal.pgen.1006317)
Supplement: S3 Table — (PDF) [file pgen.1006317.s014.pdf]

Supplementary Table S3: Centromere and neocentromere calls with Centurion algorithm

			WT Centurion Library		YJB10777 Centurion Library		YJB10780 Centurion Library	
Centromere	Genomic start	Genomic stop	Centurion call (WT)	Distance from Centurion call to CEN edge (WT)	Centurion call (neocen 4.5kb)	Distance from Centurion call to CEN edge (neocen 4.5kb)	Centurion call (neocen 166kb)	Distance from Centurion call to CEN edge (neocen 166kb)
CEN1	1562983	1565911	1564249	0	1564347	0	1563273	0
CEN2	1927141	1930100	1930097	0	1928709	0	1929645	0
CEN3	823238	826384	826605	221	824388	0	825641	0
CEN4	992531	996168	995997	0	994837	0	994630	0
CEN5	468726	471753	471356	0	5000	0	170292	0
CEN6	979972	983725	978028	1944	979794	178	979923	49
CEN7	425811	428711	427642	0	427527	0	427813	0
CENR	1743086	1747562	1743871	0	1744538	0	1743166	0
